# Does Diabetes Influence the Microbiological Response to Non-Surgical Periodontal Therapy? A Systematic Review

**DOI:** 10.3390/dj14100617

**Published:** 2026-09-23

**Authors:** Sriya Talapaneni, Lisa Jamieson, Sonia Nath

**Affiliations:** School of Dentistry, College of Health, Adelaide University, Adelaide, SA 5000, Australia

**Keywords:** periodontitis, diabetes mellitus, periodontal debridement, microbiome, periodontal pocket, periodontal attachment loss

## Abstract

**Background/Objectives**: The aim of this systematic review was to synthesise evidence on how non-surgical periodontal therapy (NSPT) alters the oral microbiota and periodontal parameters in patients with diabetes and periodontitis compared with non-diabetic patients with periodontitis. **Methods**: Electronic searches of MEDLINE/PubMed, Scopus, Embase, Ovid Emcare, JBI EBP, Web of Science, Dentistry and Oral Sciences Source, and grey literature sources were conducted from inception to March 2025. Eligible studies were clinical studies that included participants with diabetes and periodontitis, used NSPT as the primary intervention, and reported pre- and post-treatment oral microbiological and periodontal parameters as outcomes. The risk of bias was assessed using the Risk of Bias 2 (RoB 2) tool for randomised trials, and ROBINS-I for non-randomised studies and certainty of evidence was evaluated with GRADE. The review protocol was registered with PROSPERO (CRD420251005668). **Results**: Fourteen studies involving 374 participants with diabetes and 276 non-diabetic participants with periodontitis were eligible for inclusion. Thirteen studies used targeted methods restricted to pre-specified taxa, and one used 16S rRNA gene sequencing. In the targeted studies, NSPT was associated with reductions in periodontal pathogens, particularly *Porphyromonas gingivalis*, *Tannerella forsythia*, and *Aggregatibacter actinomycetemcomitans*, which were found in both diabetic and non-diabetic groups, with small or no differences observed between the groups. There were improvements in probing depth, clinical attachment, bleeding on probing and plaque scores after NSPT in both groups, with no consistent between-group differences. In most studies, RoB 2 was rated “some concerns”, and GRADE ranged from very low to moderate for microbiological and clinical outcomes. **Conclusion**: NSPT reduces selected periodontal pathogens and improves periodontal parameters in adults with periodontitis, with and without diabetes. Community-level microbiome evidence is limited to one small study, and the available data do not show whether diabetes modifies the microbiological response to NSPT.

## 1. Introduction

Periodontitis is a chronic, multifactorial inflammatory disease characterised by progressive, irreversible loss of attachment and alveolar bone [1]. Periodontitis is one of the most prevalent oral diseases, affecting 20–50% of the global population in both developed and developing countries, with a high prevalence in adolescents, adults and older individuals [2]. Periodontal disease is associated with many risk factors, such as smoking and systemic conditions, such as diabetes mellitus [3]. Individuals with diabetes mellitus are two- to three-fold more susceptible to periodontitis, and severe periodontitis can negatively impact glycaemic control, suggesting a bidirectional relationship [4]. Diabetes may contribute to periodontitis by increasing inflammatory cytokine secretion, leading to increased periodontal tissue breakdown [4]. Conversely, periodontitis results in the upregulation of the inflammatory system and thus leads to insulin resistance, exacerbating diabetes [4].

The oral microbiome plays a fundamental role in maintaining oral and systemic health, functioning as a complex ecosystem linked to various physiological processes, including host immunity, nutrient metabolism, and resistance to pathogenic organisms [5]. In periodontal health, this ecosystem is dominated by diverse commensal bacterial communities, predominantly Gram-positive, that support periodontal homeostasis and modulate the inflammatory response [6]. However, periodontal disease is associated with a dysbiotic microbial shift from health-associated commensal bacteria to increased numbers of Gram-negative periodontal pathogens such as *Porphyromonas gingivalis* (*P. gingivalis*), *Treponema denticola* (*T. denticola*) and *Tannerella forsythia* (*T. forsythia*), which are members of the ‘red complex’ that appears later in the oral biofilm development and comprises species that are considered periodontal pathogens [7,8]. These organisms possess virulence factors, including lipopolysaccharides, proteases, and immune-evasive mechanisms, that disrupt epithelial barriers, modulate host immune responses, and contribute to connective tissue breakdown and alveolar bone loss [7]. This dysbiosis not only underpins the development and progression of periodontal disease but may also influence systemic health [5]. In patients with diabetes, microbial dysbiosis is often more pronounced, with the literature reporting increased abundance of *Porphyromonas*, *Prevotella*, and *Fusobacterium* species compared with non-diabetic individuals [9].

The composition of the subgingival biofilm alone does not determine whether periodontitis develops or how rapidly it progresses. Contemporary models of pathogenesis hold that the dysbiotic biofilm is necessary but not sufficient, and that the greater part of the tissue destruction is mediated by the host inflammatory–immune response to that biofilm rather than by the microorganisms directly [10]. Current syntheses frame these inherited and acquired influences as an individual immune response that determines whether the host sustains homeostasis with the resident microbiota or fails to resolve inflammation, the latter permitting a dysbiotic community to persist and destruction to continue [11]. This host-centred perspective is directly relevant to the present review, because diabetes is among the most important acquired modifiers of that response. These concepts are reflected in the 2018 classification of periodontal and peri-implant diseases and conditions, which replaced the previous chronic and aggressive categories with a single disease entity characterised by stage, reflecting severity and anticipated complexity of management, and by grade, reflecting evidence of or risk for rapid progression [12]. Diabetes is incorporated directly into the grading framework as a modifier. Diabetes is therefore not merely a coexisting condition but a formally recognised determinant of periodontal disease trajectory, which makes the response of the subgingival microbiota to therapy in this population a question of direct clinical consequence.

Non-surgical periodontal treatment (NSPT), consisting of scaling and root planing (SRP), improves glycaemic control [13] and promotes shifts in the oral microbiota by increasing microbial diversity in Type 1 (T1DM) and Type 2 Diabetes Mellitus (T2DM) [14]. However, it remains unclear whether these microbiological changes following NSPT differ between diabetic and non-diabetic patients with periodontitis. Current work in this field characterises not only the abundance of individual periodontal pathogens but also community-level properties: alpha diversity within a sample, beta diversity between samples, overall community structure, and the ecological interactions and co-occurrence relationships that determine whether a microbial community is resilient or liable to revert after therapy. Almost none of this evidence exists for adults with diabetes and periodontitis. The great majority of available studies report targeted detection of a small number of pre-specified taxa by culture, hybridisation or PCR, methods that quantify named organisms but cannot describe community structure or ecological interaction [15].

A search of relevant databases found no current or proposed systematic reviews on the effect of NSPT on the oral microbiome in patients with diabetes, despite well-established evidence that diabetes alters host immunity, inflammatory responses [4] and microbial composition [9]. Previous reviews have examined the broader relationship between diabetes and periodontal health, including periodontal treatment [8,13], systemic outcomes [16], and glycaemic levels [17]. However, none have specifically synthesised microbiological outcomes or characterised how NSPT influences microbial shifts in diabetic versus non-diabetic individuals. This gap is significant, as understanding how NSPT affects the oral microbiome in diabetic patients may provide insight into differences in treatment response, microbial resilience, and periodontal healing. Accordingly, this systematic review aims to address this evidence gap. The research question was framed using the population (P), intervention (I), control (C), outcome (O) and study design (S) (PICOS) format: In adults with periodontitis and diabetes mellitus (P), what is the effect of non-surgical periodontal therapy (I) on the oral microbiome and clinical periodontal outcomes (O) compared with non-diabetic adults with periodontitis receiving comparable NSPT (C), as assessed in clinical studies (S)? The objectives are to: (1) identify changes in the oral microbiota following NSPT, distinguishing targeted pathogen measures from community-level microbiome measures and to determine the extent to which community-level microbiome outcomes, including diversity metrics and ecological network measures, have been reported in this population, and to characterise the resulting gap in the evidence base; (2) identify the changes in periodontal outcomes in patients with diabetes mellitus and periodontitis compared with non-diabetic patients with periodontitis following NSPT.

## 2. Methods

The systematic review followed the Preferred Reporting Items for Systematic Reviews and Meta-Analyses (PRISMA) guidelines to ensure transparent and comprehensive reporting throughout the process (Supplementary Table S1). The review protocol was registered with PROSPERO (registration ID: CRD420251005668).

### 2.1. Eligibility Criteria

Studies were selected according to pre-specified eligibility criteria defined using the PICOS framework (Supplementary Table S2).

#### 2.1.1. Population

Adults with periodontitis and either T1DM or T2DM were included, with no restrictions on age, sex, language or geography, provided a comparison group of non-diabetic individuals with periodontitis was available. Both T1DM and T2DM were eligible, and studies enrolling both were included, provided results were reported for a diabetic group as a whole or by type. Diagnosis of diabetes: a physician-recorded diagnosis, current use of glucose-lowering medication, or a laboratory diagnosis reported by the study authors was accepted; a specific HbA1c or fasting glucose threshold was not imposed. Glycaemic control was recorded where reported but was not an eligibility criterion. Definition of periodontitis: the case definition applied by the original authors was accepted, reflecting the range of classifications in use across the publication period; no minimum probing depth or attachment loss threshold was imposed. Although much of the evidence linking diabetes to periodontitis derives from T2DM, T1DM is also associated with greater periodontal destruction, and this association is detectable in children and adolescents [18]. Susceptibility to periodontitis is increased approximately threefold in people with diabetes, and the degree of hyperglycaemia rather than the type of diabetes appears to be the principal determinant of periodontal severity.

#### 2.1.2. Intervention

The intervention was non-surgical periodontal therapy (NSPT), defined as scaling and root planing (SRP) with or without adjunctive measures, delivered to patients with diabetes and periodontitis; the comparison group comprised periodontitis patients without diabetes who received comparable NSPT. NSPT protocols included subgingival mechanical debridement delivered quadrant-wise or as full-mouth debridement, with or without adjunctive antimicrobial or other agents, provided the protocol was applied comparably to the diabetic and non-diabetic groups within the study. Follow-up of at least one post-treatment microbiological assessment was required and no minimum duration was imposed.

Studies in which mechanical debridement was supplemented by an adjunctive agent were eligible for inclusion. Because the review question concerned whether the response to NSPT differs between people with and without diabetes, eligibility required only that the treatment protocol, including any adjunct, be delivered under the same protocol to the diabetic and non-diabetic arms within a given study. Restricting eligibility to mechanical debridement alone would have excluded a substantial proportion of the available evidence.

#### 2.1.3. Comparator

The control group included patients with periodontitis but without T1DM or T2DM who also underwent NSPT.

#### 2.1.4. Outcomes

Primary outcomes were oral microbiological changes (bacterial counts, percentages of sites colonised, species proportions, relative or differential abundances, and alpha and beta diversity) in diabetic and non-diabetic periodontitis patients. No restriction was placed on the detection method; culture, hybridisation, PCR-based, and sequencing-based studies were all eligible, since restricting to sequencing would have left too few studies to address the review question.

Secondary outcomes were changes in clinical periodontal parameters, including probing depth (PPD) [19], clinical attachment loss (CAL) [20], bleeding on probing (BOP) [21], and plaque levels, measured as presence/absence, full-mouth plaque scores or plaque index [22]. In terms of the periodontitis terminology and classification current at the time they were conducted, describing participants as having chronic periodontitis of mild, moderate or severe extent or under the 2018 classification, the same cases would be reported as periodontitis, staged I to IV by severity and complexity, and graded A to C by progression risk [12]. The classification system, case definition, inclusion threshold and reported severity of each study were extracted as reported by the original authors. The original terminology has therefore been retained for fidelity to the source reports. Under the current criteria, the diabetic participants in these studies would be graded B or C based on diabetes status alone [12].

#### 2.1.5. Study Designs

Randomised controlled trials, non-randomised clinical trials, and prospective and retrospective cohort studies were eligible. Studies without a non-diabetic comparison group, case reports, case series, narrative and systematic reviews, and in vitro and animal studies were excluded.

### 2.2. Search Strategy

This review employed a three-stage search strategy. An initial electronic search was conducted in MEDLINE/PubMed to identify all potentially relevant articles on the oral microbiome in patients with periodontitis and diabetes mellitus following non-surgical periodontal therapy, from the inception of the platform through March 2025. In the pilot search, all relevant terms (i.e., controlled-vocabulary terms and text words) were identified, and the main search strategy was developed. Second, a comprehensive search was conducted across Scopus, Embase, Ovid Emcare, the Joanna Briggs Institute Evidence-Based Practice (JBI EBP) Database, Web of Science, and Dentistry and Oral Sciences Source (DOSS) (Supplementary Table S3). Keywords included “oral microbiome,” “periodontitis,” “diabetes mellitus,” “non-surgical periodontal therapy,” and related terms. Third, a reference list of all selected articles was compiled, and an additional freehand literature search of the bibliographic index of these articles was conducted.

A systematic approach to grey literature was developed to identify all relevant reports using OpenGrey, ProQuest, and relevant conference proceedings. Review authors conducted an online web search using a combination of keywords, such as “oral microbiology”, “periodontitis diabetes” and “non-surgical periodontal therapy”. In addition, Google Scholar was searched to identify potentially relevant studies and minimise publication bias. A search using the same key terms was conducted, and the first 10 pages of results (approximately 100 records, sorted by relevance) were screened by two reviewers (ST and SN). Any disagreements were resolved by the third author (LMJ). Furthermore, expert authors in the field were contacted via email for additional unpublished data.

### 2.3. Study Selection

Studies identified through the database searches were first imported into EndNote 21 (Clarivate Analytics, PA, USA) for citation management. The de-duplicated library was then exported to Covidence, an online systematic review platform used to organise screening, full-text review, and data extraction. The study selection was carried out in two steps: title and abstract screening and full-text screening. Using this software, two independent reviewers (ST and SN) screened the titles, abstracts, and full texts of the selected articles against predefined inclusion criteria. All excluded full-text articles were documented with a specific reason for exclusion. Any disagreements at any stage were resolved through discussion with a third reviewer (LMJ).

### 2.4. Data Extraction

A customised data extraction form was developed, pilot-tested, and refined for this review. Two calibrated reviewers (ST and SN) extracted data independently, with a third reviewer (LMJ) consulted to resolve discrepancies. When information was missing or unclear, we contacted corresponding authors via email to obtain additional data.

The data extraction form captured the following domains: (1) article information; (2) study characteristics; (3) participant characteristics including diabetes type, the criterion used to diagnose diabetes, baseline and post-treatment HbA1c, glycaemic control categories and thresholds, diabetes duration and glucose-lowering therapy, and the periodontitis classification system, case definition and severity; (4) details of the NSPT or any adjunctive treatment, including the delivery format (full-mouth or quadrant-wise); (5) oral microbiological outcomes, together with the detection method and sampling site; and (6) clinical periodontal outcomes, including measures of variability such as standard deviations or confidence intervals when available. Where necessary, standard methods recommended in the Cochrane Handbook were applied to derive missing dispersion estimates from reported confidence intervals or other summary statistics. For studies that reported results only in graphical form (e.g., bar or column charts), numerical data were extracted using WebPlotDigitizer (Version 4.2, Automeris LLC, GNU Affero General Public License, TX, USA), a computer-vision-assisted digitising tool, to maximise completeness and accuracy.

### 2.5. Risk of Bias Assessment and Certainty of Evidence

All included articles were assessed in full, including their methodological quality. RCTs were evaluated using the revised Cochrane Risk of Bias tool (RoB 2) [23]. Non-randomised studies were appraised using the Risk of Bias in Non-randomised Studies of Interventions tool (ROBINS-I) [24] with respect to overall risk-of-bias judgments (low, some concerns, or high for RoB 2; low, moderate, serious, or critical for ROBINS-I). The certainty of the body of evidence for each outcome was assessed using the Grading of Recommendations Assessment, Development and Evaluation (GRADE) approach [25]. The final ratings (high, moderate, low, or very low certainty) were summarised in evidence profiles and used to guide the interpretation of the review findings. Two reviewers (ST and SN) independently performed the assessments and resolved disagreements through discussion or consultation with a third reviewer (LMJ).

### 2.6. Data Synthesis and Analysis

Following data extraction, study characteristics and outcomes were tabulated in Microsoft Excel to assess clinical and methodological comparability. A meta-analysis was planned if the studies were sufficiently homogeneous in design, populations, interventions, and outcome measures. The feasibility of quantitative synthesis was assessed outcome by outcome, as described below, and a synthesis without meta-analysis was undertaken otherwise.

Quantitative synthesis was considered for each outcome and was judged feasible only if three conditions were met: (i) at least three studies reported the same taxon or clinical parameter on a common or convertible scale, from a comparable sampling site and at a comparable follow-up time; (ii) group-level estimates of change with a measure of dispersion were available for both the diabetic and the non-diabetic group; and (iii) the studies were sufficiently similar, clinically and methodologically, for a pooled estimate to be interpretable.

For microbiological findings, studies were grouped according to two dimensions. The first was the analytical level of the microbiological assessment. Targeted pathogen analyses were defined as methods that detect or quantify a restricted, pre-specified set of taxa: culture, species-specific or quantitative PCR, the rapid urease test, and checkerboard DNA–DNA hybridisation with a closed panel of probes. Community-level (microbiome) analyses were defined as open-ended, sequencing-based methods that characterise the whole detectable community without a pre-specified target list and therefore permit estimation of alpha and beta diversity, community composition and co-occurrence structure. Findings at the two levels are reported separately and the term ‘microbiome’ is used in this review only for community-level evidence. To visualise the direction and consistency of microbiological change across studies, each study-taxon observation was coded as an increase, no change, or decrease in the reported abundance, count, proportion or prevalence of that taxon from baseline to follow-up, separately for diabetic and non-diabetic participants. Direction was taken from the original authors. Because the included studies reported at differing taxonomic depths, observations were grouped into three levels of resolution: microbial complex, genus, and species. The coded observations were displayed as bubble plots generated in R (version 4.4.2; R Foundation for Statistical Computing, Vienna, Austria) using the ggplot2 package.

The second dimension was the treatment protocol: mechanical debridement alone, or mechanical debridement combined with an adjunctive agent.

## 3. Results

### 3.1. Search Results and Study Selection

A PRISMA 2020 flow diagram summarises the study selection process (Figure 1). Database and register searches identified 7921 records, of which 4067 were removed before screening (3316 duplicate records and 751 records marked as ineligible by automation tools), leaving 3854 records for title and abstract screening. Of these, we excluded 3800 records, and we sought 54 reports for retrieval. One report could not be retrieved for eligibility assessment [26], leaving 53 articles for full-text screening. Following full-text screening, a further 39 articles were excluded (Supplementary Table S4). A further 34 records identified through Google Scholar were assessed at full text, and all were excluded. Fourteen studies met the inclusion criteria and were included in the review [27,28,29,30,31,32,33,34,35,36,37,38,39,40].

### 3.2. The Main Characteristics of the Included Studies

Table 1 describes the studies included in the review. Three studies were RCTs [27,31,38], five were prospective studies [29,32,35,36,40], three were cohort studies [30,34,37], and three were clinical trials [28,33,39], all published between 1998 and 2024. Included studies spanned diverse countries of origin, with four studies from Brazil [27,30,32,39], four studies from India [34,36,37,38], and a single study from Serbia [35], Slovenia [33], China [40], Germany [29], Malaysia [28] and Indonesia [31]. Across all studies, 374 participants with diabetes and periodontitis and 276 non-diabetic participants with periodontitis were included. Eleven studies included participants with T2DM only [27,30,31,32,34,35,36,37,38,39,40]; one study included participants with T1DM only [28]; and two studies included participants with both T1DM and T2DM [29,33]. Supplementary Table S5 summarises diabetes characteristics, and baseline HbA1c was reported for the diabetic group in 11 of the 14 studies and for the non-diabetic group in eight; where reported, mean baseline HbA1c in the diabetic groups ranged from 6.15 to 9.43%. Two studies stratified participants with diabetes by glycaemic control [34,35] and one enrolled only participants with inadequate metabolic control [39], using thresholds that differed between studies; one study stratified participants by diabetes type [33], and the remaining studies reported a diabetes diagnosis without glycaemic stratification. Eight studies reported diabetes duration and glucose-lowering therapy. Supplementary Table S6 shows periodontal diagnostic criteria, disease severity, and treatment protocols. Participants with chronic periodontitis were classified as mild [34], moderate [28,29,34,37,39] or severe/advanced [28,29,37,39]. Two studies applied the 2018 World Workshop classification [31,33]. Participants could not be re-staged or re-graded under the 2018 classification because individual-level data were not available. The most common type of intervention was NSPT, consisting of full-mouth scaling and root planing (FMSRP). Some studies also incorporated adjunctive antibiotic therapy [32], chlorhexidine irrigation [27,29,33], chlorhexidine rinses [27,33] and hyaluronic acid gel [31]. The included studies are grouped here by treatment protocol. Nine studies delivered mechanical debridement alone [28,30,34,35,36,37,38,39,40]. Duarte et al. [32] used NSPT with adjunctive systemic antibiotics. Almeida et al. [27], Christgau et al. [29], and Hropot Plesko et al. [33] used NSPT with adjunctive chlorhexidine as subgingival irrigation, as a rinse, or as both. Devina et al. [31] used NSPT with adjunctive hyaluronic acid gel and [31] included a placebo gel arm, allowing the mechanical-therapy-only comparison to be recovered. Full-mouth scaling and root planing rather than quadrant-wise was the most commonly described delivery format. Because only one or two studies fall into each adjunctive category, a formal subgroup analysis by protocol was not feasible.

### 3.3. Microbiological Outcomes: Targeted Pathogen Analyses and Community-Level Analysis

The taxa were reported as culture counts, genome copies, relative expression, proportions of total DNA, or the percentage of positive sites or patients. These metrics measure different constructs (bacterial load versus prevalence) rather than the same construct in different units, so conversion to a standardised mean difference would have combined different quantities. Sampling sites and follow-up times (two weeks to twelve months) also differed, and no combination of taxon, metric, sampling site and follow-up time was reported by more than three studies. Among the 14 included studies (Table 2), microbiological samples were collected from saliva [27,37], subgingival biofilm/plaque [28,29,30,31,32,33,35,36,38,39], or both locations [40]. The microbiological methods used across the included studies fall into four categories, and results should be read with the category in mind because the methods are not interchangeable. Thirteen of the fourteen studies therefore provide targeted pathogen data only. Culture-based detection on selective or non-selective agar was used [29], along with checkerboard DNA-DNA hybridisation, which detects a fixed panel of species and reports counts or proportions [32,39]. PCR-based detection of pre-specified target species was the most common approach and was used by most studies [27,28,31,33,34,35,36,37,41]. Sambashivaiah et al. [38] used a rapid urease test. A single study [40] applied sequencing-based characterisation using 16S rRNA gene amplicon sequencing; consequently, it is the only source of diversity and community-structure outcomes in this review. Culture and hybridisation methods quantify a restricted panel of organisms, PCR methods quantify only the targets selected in advance, and only sequencing can describe the community as a whole; comparisons of absolute values across these categories are therefore not meaningful, and the synthesis in this review is restricted to the direction of change within each study. Each study collected microbial samples at baseline and in at least one follow-up interval, though the exact timing of follow-up measurements varied across different studies and ranged from two weeks to twelve months.

#### 3.3.1. Targeted Pathogen Analyses

At baseline, *P. gingivalis*, *T. forsythia* and *A. actinomycetemcomitans* were the most consistently reported periodontal pathogens across the included studies, with most studies identifying high levels of these species in both diabetic and non-diabetic participants with periodontitis. A reduction in *P. gingivalis* after NSPT was the most consistently reported microbiological finding. Buzinin et al. [28], Hropot Plesko et al. [33], Rode et al. [37] da Cruz et al. [30], and Mirnić et al. [35] found significant reductions in non-diabetic participants and those with diabetes at three months. Devina et al. [31] reported a significant decrease in *P. gingivalis* at four weeks in both non-diabetic and diabetic participants treated with NSPT and a placebo gel, while the reduction in participants receiving adjunctive hyaluronic acid gel was non-significant. Almeida et al. [27] reported non-significant reductions at six months, and Hropot Plesko et al. [33] (T2DM group) at three months. Christgau et al. [29] and Lakhani et al. [34] also reported non-significant decreases at four months. Significant reductions in *T. forsythia* were reported by multiple studies. Almeida et al. [27] found a significant decrease in the frequency of *T. forsythia* in diabetic participants at six months post-NSPT compared with the non-diabetic group. Buzinin et al. [28], Hropot Plesko et al. [33] and Mirnić et al. [35] reported a decrease in both diabetic and non-diabetic participants at three months. Several studies reported reductions in *A. actinomycetemcomitans* following NSPT. Hropot Plesko et al. [33] found a significant reduction among participants with T1DM at three months post-NSPT, while Mirnić et al. [35] reported a significant reduction at three months in both non-diabetic participants and those with poorly controlled diabetes, and a non-significant decrease in participants with well-controlled diabetes. Several studies reported on additional species. Rajpurohit et al. [36] found a significant reduction in *Mogibacterium timidum* counts at six weeks post-NSPT across all three groups (non-periodontitis, non-diabetic with periodontitis and diabetic with periodontitis). Mirnić et al. [35] reported a significant reduction in *P. intermedia*. Sambashivaiah et al. [38] found a significant reduction in the prevalence of *Helicobacter pylori* at two weeks post-NSPT in both non-diabetic and diabetic participants. Duarte et al. [32] reported a significant reduction in the proportions of red complex bacteria. Overall, in the targeted analyses, NSPT was followed by reductions in key periodontal pathogens (especially red-complex species) in both diabetic and non-diabetic participants, with most studies showing similar directional shifts and only isolated between-group differences (e.g., slightly greater *T. forsythia* reduction in diabetics [27], larger *P. gingivalis*, *T. forsythia* reduction in diabetics [28], stronger *A. actinomycetemcomitans* change in T1DM) [33]. Overall, diabetics tended to retain somewhat higher pathogen loads or complex proportions in a few studies [32,36], but there was no consistent microbiological advantage for either group. These between-group differences were each reported by a single study, were not consistent in direction and, with 10 to 90 participants with diabetes per study, should be regarded as hypothesis-generating; the similarity in the direction of change indicates that both groups responded, not that the magnitude of response was equivalent. Figure 2 displays the direction of change for every taxon observation in non-diabetic (Figure 2A,B) and diabetic (Figure 2C,D) participants. Decreases predominated at the species level in both groups, with *P. gingivalis*, *T. forsythia*, *A. actinomycetemcomitans*, *P. intermedia* and *T. denticola* the most frequently reported taxa. Both groups showed increases in *Actinomyces* species and *E. corrodens*. Discordance was limited to a small number of taxa reported by single studies. Because all but one of the studies contributing to Figure 2 used targeted methods, the figure summarises change in pre-specified taxa and should not be read as a representation of community-level change.

The included studies characterised diabetes status inconsistently, which affects how to interpret these microbiological findings (Supplementary Table S5). Where stratification was performed, glycaemic control appeared to modify the microbiological response. Mirnić et al. [35] reported significant reductions in *A. actinomycetemcomitans* at three months in non-diabetic participants and in those with poorly controlled diabetes, but only a non-significant decrease in participants with well-controlled diabetes. Hropot Plesko et al. [33] separated participants by diabetes type rather than by glycaemic control, reporting a significant reduction in *A. actinomycetemcomitans* in the T1DM group and a non-significant reduction in *P. gingivalis* in the T2DM group. Most reports did not report diabetes treatment, including whether participants received insulin, oral hypoglycaemic agents, or dietary management alone. These observations are presented because glycaemic control is a plausible modifier of the microbiological response to therapy; however, the small number of stratified studies and the absence of a common HbA1c threshold mean that the present evidence does not support any conclusion about its influence.

#### 3.3.2. Community-Level Microbiome Analysis

Only one study, Wang et al. [40], assessed the microbiota at the community level, using 16S rRNA gene amplicon sequencing of subgingival plaque and saliva. Alpha diversity (observed features, Shannon and Simpson indices) and beta diversity (UniFrac distances) did not differ between the diabetic and non-diabetic groups. In participants with diabetes, NSPT was followed by increased subgingival diversity, decreased relative abundance of *Porphyromonas*, *Filifactor*, and *Eubacterium*, and increased genera including *Streptococcus*, *Actinomyces*, *Veillonella*, and *Leptotrichia*; co-occurrence network analysis suggested a denser post-treatment network in the diabetic group. Because this is a single study of 22 participants, with a three-month follow-up and a serious risk of bias under ROBINS-I, they do not establish that microbial diversity, community structure or ecological stability respond differently to NSPT in people with diabetes, and no synthesis of community-level outcomes was possible.

#### 3.3.3. Microbiological Findings by Treatment Protocol

In every mechanical-only study that measured *P. gingivalis* or *T. forsythia* [28,30,31,34,35,37,39], these taxa decreased from baseline in both the diabetic and the non-diabetic groups; the only exception was an unchanged prevalence of *T. forsythia* at sites with PPD ≥ 5 mm in the diabetic group of da Cruz et al. [30]. The two remaining mechanical-only studies that used targeted methods, which measured *M. timidum* [36] or *H. pylori* [39], likewise reported reductions in both groups. Christgau et al. [29] reported decreases in *P. gingivalis* in both groups, and red-complex proportions decreased in both groups after adjunctive antibiotics, although diabetic participants retained higher red- and orange-complex proportions at 12 months [32]. In contrast, salivary *P. gingivalis* levels exceeded baseline values in both groups at 12 months in Almeida et al. [27]; *P. gingivalis* increased in the diabetic group receiving hyaluronic acid gel, whereas it decreased in the corresponding placebo arm [31]; and in Hropot Plesko et al. [33], *P. gingivalis* prevalence was unchanged in the T1DM group, and *T. forsythia* prevalence was unchanged in the T2DM group. Because each adjunct was represented by only one to three studies, the contribution of the adjunct cannot be separated from other study-level differences in population, sampling and detection method. Restricting the synthesis to mechanical debridement alone did not, however, change the principal finding that targeted pathogens decreased in both diabetic and non-diabetic participants.

### 3.4. NSPT Was Associated with Improved Periodontal Clinical Outcomes in Both Diabetic and Non-Diabetic Participants

The clinical outcomes assessed across the included studies were PPD, CAL, BOP and plaque scores. PPD and CAL were reported more uniformly, and a meta-analysis of the difference in change between the diabetic and non-diabetic groups was considered. It was not undertaken because this estimate requires the standard deviation of the change from baseline, or the baseline–follow-up correlation, which was rarely reported; because site selection (full-mouth versus deepest sites) and follow-up intervals varied; and because treatment protocols differed in their use of adjuncts. A synthesis without meta-analysis was therefore undertaken and is reported in accordance with the Synthesis Without Meta-analysis (SWiM) guideline [42], using the direction of change as the standardised metric.

PPD (Supplementary Table S7) was reported in 11 studies [27,28,29,30,31,32,33,35,37,39,40] at baseline and at follow-up intervals ranging from 2 weeks to 12 months post-NSPT. All studies that reported mean PPD found reductions in both diabetic and non-diabetic participants at the first or second post-treatment interval. Across most studies, the magnitude of PPD reduction was broadly similar between non-diabetic and diabetic participants. Duarte et al. [31], Hropot Plesko et al. [33], Mirnić et al. [35] and Silva-Boghossian et al. [39] reported comparable reductions across groups; Rode et al. [37] and Wang et al. [40] observed a slightly greater decrease in the non-diabetic group. Conversely, Almeida et al. [27], Buzinin et al. [28] and Christgau et al. [29] reported a marginally more pronounced decrease in the diabetic group at most follow-up intervals. Similarly, CAL (Supplementary Table S8) was reported in the same 11 studies as PPD [27,28,29,30,31,32,33,35,37,39,40], over comparable time intervals (2 weeks to 12 months). All studies found reductions in CAL in both groups from baseline, although two studies reported a transient increase at an intermediate interval. Most studies reported broadly similar reductions across groups.

BOP (Supplementary Table S9) was assessed in six studies [27,28,31,32,33,39] at follow-up intervals ranging from 4 weeks to 12 months. All six studies reported a reduction in BOP as a percentage of probing sites at all post-NSPT time points compared to baseline in both groups. Plaque accumulation (Supplementary Table S10) was assessed across several studies using a range of indices, including the percentage of sites with plaque accumulation and the PI [27,28,30,32,33,35,40]. Most studies reporting plaque measures found substantial reductions in plaque scores following NSPT in both diabetic and non-diabetic participants, with improvements evident at each follow-up interval compared to baseline. No study reported an increase in plaque scores relative to baseline at any follow-up point.

Taken together, the clinical evidence consistently demonstrates that NSPT improves PPD, CAL, BOP, and plaque scores in both diabetic and non-diabetic adults with periodontitis. While most studies found similar improvements across groups, a subset reported marginally greater reductions in either non-diabetic or diabetic participants, without a consistent directional pattern. These findings suggest that diabetes does not preclude meaningful clinical improvement following NSPT, although glycaemic control and disease severity may influence the degree of benefit.

### 3.5. Risk of Bias and GRADE Assessment

Table 1 and Supplementary Figures S1 and S2 summarise the risk-of-bias assessments. Six were assessed with the RoB 2 tool, and eight were assessed using the ROBINS-I tool. Three studies [34,37,40] assessed using the ROBINS-I tool were deemed to have a serious risk of bias because one or more main aspects of the bias assessment were serious.

The GRADE ranged from very low to moderate across microbiological and clinical outcomes (Supplementary Table S11). RCTs generally provided higher-certainty evidence than observational studies; however, overall certainty for most microbiological outcomes was downgraded because of risk of bias, inconsistency across studies, imprecision from small sample sizes, and heterogeneity in outcome measurement. For clinical periodontal outcomes, certainty was typically higher but was still limited by similar concerns about risk of bias and inconsistency.

## 4. Discussion

This systematic review showed that, in studies using targeted detection methods, NSPT was consistently associated with reductions in key periodontal pathogens, particularly *P. gingivalis*, *T. forsythia* and *A. actinomycetemcomitans*, in both diabetic and non-diabetic adults with periodontitis, alongside improvements in PPD, CAL, BOP and plaque scores. These findings indicate that people with diabetes show within-group microbiological and clinical improvement after NSPT. They do not indicate whether that improvement is larger than, smaller than, or equivalent to that seen in people without diabetes, and the available studies could not determine whether the response varies with glycaemic control, baseline microbial load, or adjunctive therapy.

The review question implies an effect-modification hypothesis: that the microbiological response to NSPT differs by diabetes status. Testing that hypothesis requires comparing change from baseline between diabetic and non-diabetic groups (a diabetes-by-time interaction), ideally accounting for baseline differences in disease severity and pathogen load. The included studies do not permit this. Most reported within-group change relative to baseline; between-group comparisons of change were reported inconsistently and were based on 10 to 90 participants with diabetes per study, too few to detect a modest difference in response; and no pooled between-group estimate could be derived. The review therefore demonstrates that NSPT is followed by reductions in targeted periodontal pathogens and by clinical improvement in both populations. It does not show that diabetes modifies the treatment response, nor does it show that the response is equivalent. The broadly similar direction of change should be read as an absence of evidence of a difference rather than as evidence that no difference exists, and the isolated between-group differences reported are best regarded as hypothesis-generating. An adequately powered comparative study with a pre-specified test of the diabetes-by-treatment interaction, standardised microbiological methods and concurrent HbA1c measurement would be needed to answer the review question.

Several methodological limitations in the included evidence warrant acknowledgment before drawing firm conclusions. Variation in adjunctive therapy contributes to heterogeneity. Most studies delivered mechanical debridement alone but supplemented it with systemic antibiotics, chlorhexidine irrigation or rinsing, or hyaluronic acid gel. Because only one to three studies fall into each adjunctive category, subgroup analysis by protocol was not feasible; however, when the synthesis was restricted to mechanical debridement alone, targeted pathogens still decreased in both diabetic and non-diabetic groups, indicating that the principal finding is not attributable to the adjunctive studies. The separate contribution of each adjunct could not be assessed. Trials comparing diabetic and non-diabetic participants under a standardised debridement-only protocol are needed to resolve this. The included studies were highly heterogeneous in microbiological methods, and outcomes were expressed as bacterial counts, mean proportions, percentages of positive sites or patients, or relative abundances, all of which are difficult to compare directly. This heterogeneity contrasts with contemporary microbiome research, which increasingly favours high-throughput sequencing with standardised bioinformatic pipelines, and limits the translational value of findings from older studies that relied on culture or hybridization [15]. The predominance of non-randomised study designs, small sample sizes, and the absence of blinding in many studies contributed to a risk of bias ranging from low to serious, with three studies assessed as carrying a serious risk of bias under ROBINS-I [34,37,40]. The high degree of methodological and outcome heterogeneity precluded quantitative meta-analysis, consistent with prior periodontal systematic reviews limited to narrative synthesis due to similar analytical incompatibility [13,16]; consequently, the certainty of evidence for microbiological outcomes ranged from very low to moderate under the GRADE framework. A further limitation concerns glycaemic control specifically. Although glycaemic status is the most plausible modifier of the periodontal and microbiological response in this population, the included studies did not report it in a form that permitted analysis. HbA1c was reported inconsistently; thresholds for defining well- and poorly controlled diabetes differed between studies, and only a minority stratified microbiological outcomes by glycaemic status. Therefore, it was not possible to determine whether the microbiological response to NSPT varies with the degree of hyperglycaemia. The finding of broadly comparable responses in diabetic and non-diabetic participants should not be read as evidence that glycaemic control is unimportant to the microbiological outcome of therapy, but rather as evidence that the available studies were not designed, powered or reported in a way that would allow such an effect to be detected. Another limitation is that the follow-up intervals varied, and this range was retained because the review question concerned whether the direction of the microbiological response to NSPT differs by diabetes status rather than the durability of that response, and because within each study the diabetic and non-diabetic arms were sampled at identical intervals, so the between-group contrast is unaffected by the interval chosen. Comparison of absolute values across studies with differing follow-up periods should nonetheless be avoided. Subgingival recolonisation following mechanical debridement is well recognised, and in the absence of structured supportive care the subgingival pathogen profile moves back towards pre-treatment levels over the months following therapy [43]. Observations at the longer intervals therefore reflect the combined effect of treatment and recolonisation rather than a sustained microbiological benefit and are interpreted here accordingly.

It is also useful to position these findings against the previous syntheses in this area. Earlier systematic reviews and meta-analyses have examined the effect of NSPT on glycaemic outcomes and clinical periodontal parameters in people with diabetes and have generally reported improvements in probing depth and attachment level comparable to those seen in people without diabetes, alongside a modest reduction in HbA1c following therapy [13,16,17]. The present review differs from those syntheses in its outcome domain rather than its population: where previous work pooled clinical and metabolic endpoints, this review addresses the microbiological response, for which no pooled estimate could be derived because of the heterogeneity described above. The added value of the present work therefore lies in establishing the direction and consistency of microbiological change across both groups, and in quantifying how little community-level microbiome evidence currently exists, rather than in refining an existing pooled estimate.

The bidirectional relationship between diabetes and periodontitis is well established, and severe periodontitis independently impairs glycaemic control [4]. A key mechanism is the altered subgingival microbiome observed in diabetic individuals, in which elevated gingival crevicular fluid glucose concentrations provide a substrate for pathobiont proliferation, promoting dysbiosis and increasing the burden of Gram-negative anaerobes such as *Porphyromonas*, *Prevotella*, and *Fusobacterium* species [3,9]. This dysbiotic shift drives upregulation of pro-inflammatory cytokines, including IL-1β and TNF-α, thereby promoting connective tissue breakdown, alveolar bone resorption, and, systemically, insulin resistance, creating a self-reinforcing cycle of metabolic and periodontal deterioration [44]. The red complex species *P. gingivalis* and *T. forsythia*, reported as dominant pathogens at baseline across most included studies, are central orchestrators of periodontal dysbiosis through virulence factors such as lipopolysaccharides, gingipains and immune-evasion mechanisms that disrupt epithelial barriers and drive connective tissue and alveolar bone destruction. The targeted data in the present review are consistent with mechanical debridement reducing the burden of these key pathogens in diabetic patients: Buzinin et al. [28] and Mirnić et al. [35] demonstrated significant reductions in *P. gingivalis* and *T. forsythia* in diabetic participants, consistent with evidence that NSPT-mediated biofilm disruption reduces bacterial load, virulence factor expression and downstream inflammatory mediators; whether this reflects disruption of the dysbiotic community as a whole cannot be determined from targeted methods. However, the magnitude and statistical significance of these changes were not uniform: some diabetic subgroups, particularly those with poorly controlled glycaemia, exhibited attenuated or non-significant reductions in microbial levels compared with non-diabetic participants, as observed by Almeida et al. [27]. Impaired neutrophil function and heightened systemic inflammatory tone in poorly controlled diabetes have been proposed to limit microbial resolution after treatment, but the included studies did not examine this, and in the only study that stratified microbiological outcomes by glycaemic control, reductions in several taxa were most marked in participants with poorly controlled diabetes [35]. Importantly, Wang et al. [40] was the only study to apply diversity metrics. The near absence of sequencing outcomes in the remaining literature represents a major evidence gap, given that such measures are now standard in contemporary microbiome research and are essential for characterising community-level responses to NSPT in diabetic populations.

The within-group reductions in targeted pathogens and the clinical improvements observed in both diabetic and non-diabetic groups support the view that periodontal treatment should not be withheld from patients with diabetes; this inference rests largely on observational studies of heterogeneous design, and the evidence does not show whether the benefit depends on glycaemic control. These improvements align with the well-established evidence base supporting NSPT as the first-line intervention for periodontitis.

Three recommendations are offered, drawing on the wider evidence base [4,13,17] as well as on the present findings: integrating routine NSPT and supportive periodontal care into diabetes management pathways, ensuring that all adults with diabetes are screened for periodontitis and, where present, receive timely debridement with structured maintenance at intervals tailored to their glycaemic control and periodontal risk profile [13]; strengthening interprofessional collaboration between dental and medical teams by systematically sharing information on periodontal status and glycaemic control, optimising HbA1c before and during NSPT where feasible, and using periodontal improvement as a prompt to reinforce broader cardiometabolic risk reduction [4,13,17,45]; as well, targeted microbiological monitoring should be incorporated in specialist and research settings, using standardised sampling and contemporary sequencing to track key pathogens and diversity measures over time, thereby linking microbial shifts with clinical response and glycaemic status [40].

Standardised microbiological protocols address four elements that currently differ between studies: first, the sampling site and method, since salivary, pooled subgingival and site-specific paper-point samples are not interchangeable; second, the timing of sampling relative to therapy, with at least one early and one later time point, so that the initial response can be distinguished from subsequent recolonisation; third, the analytical platform and the reporting unit, with quantitative PCR or 16S rRNA gene sequencing reported as absolute quantities alongside relative abundance, rather than as presence or absence or as the percentage of positive sites; fourth, concurrent reporting of HbA1c at each microbiological sampling point, so that microbial change can be related to glycaemic status. The rationale is that the pathogen profile of established periodontitis is broadly similar in people with and without diabetes, so any difference attributable to diabetes is likely to emerge in the response to therapy rather than at baseline. Detecting such a difference requires post-treatment measurements to be taken in the same way, at the same time, and on the same scale across studies, which is not currently the case.

## 5. Conclusions

This systematic review found that non-surgical periodontal therapy was followed by reductions in targeted periodontal pathogens and by clinical periodontal improvement in both diabetic and non-diabetic adults with periodontitis. These findings rest on very low- to moderate-certainty evidence from small, heterogeneous studies. Community-level microbiome evidence is limited to a single small study, so no conclusion can be drawn about the effect of NSPT on microbial diversity, community structure or ecological stability in people with diabetes.

### 5.1. Implications for Clinical Practice

First, the available evidence suggests that NSPT is associated with clinically meaningful periodontal improvement in both groups, potentially reducing the need for surgery and its associated infection and wound-healing risks in patients with diabetes, who are more vulnerable to postoperative complications. Second, the evidence highlights the importance of interdisciplinary collaboration between dental clinicians and medical teams, with optimisation of HbA1c prior to and during periodontal therapy likely to support host healing, although the present review could not assess whether glycaemic optimisation alters the microbiological response. Third, the findings suggest that more frequent maintenance visits, meticulous biofilm control and targeted monitoring of periodontal pathogens may be warranted in diabetic patients, given that microbial rebound was observed at longer follow-up intervals in some studies.

### 5.2. Implications for Research

Future research should incorporate standardised microbial sampling protocols, advanced sequencing approaches, robust ecological analyses, and functional metagenomics. Larger, well-controlled longitudinal studies with a pre-specified test of the diabetes-by-treatment interaction are needed to clarify whether microbial shifts differ meaningfully by diabetic control and whether microbiome changes correlate with systemic inflammatory or metabolic measures. Such studies would also enable exploration of whether specific microbial signatures can serve as biomarkers for periodontal response in diabetic populations.

## Figures and Tables

**Figure 1:**
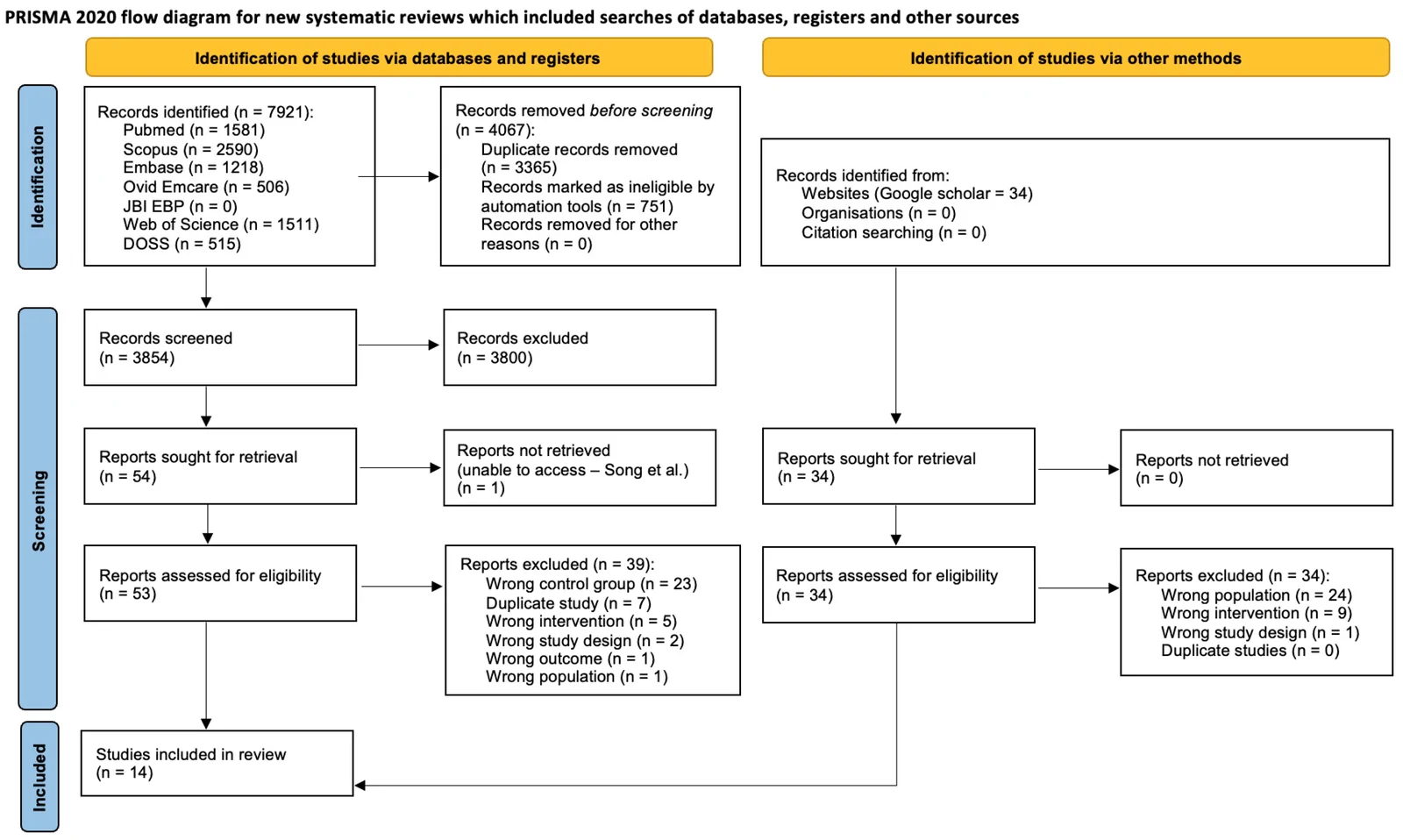
PRISMA 2020 flow diagram. Evaluating the effects of non-surgical periodontal therapy on oral microbiota and periodontal outcomes in diabetic and non-diabetic adults with periodontitis [26].

**Figure 2:**
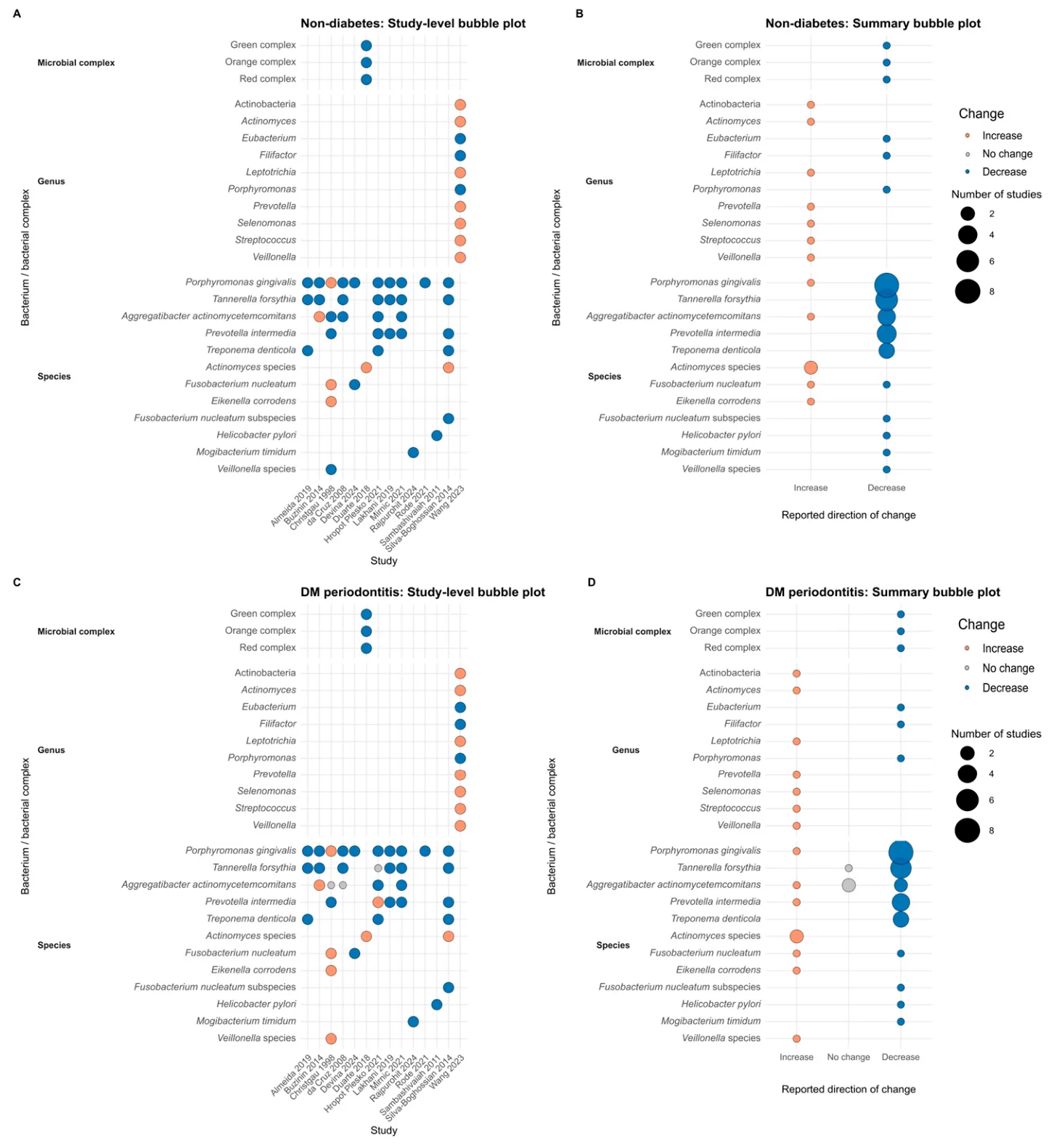
Microbiological changes following non-surgical periodontal therapy in diabetic and non-diabetic periodontitis patients [27,28,29,30,31,32,33,34,35,36,37,38,39,40]. Study-level (**A**,**C**) and summary (**B**,**D**) bubble plots showing reported post-treatment changes in individual bacterial species and complexes following non-surgical periodontal therapy in (**A**,**B**) non-diabetic adults with periodontitis and (**C**,**D**) adults with diabetes-associated periodontitis. Each bubble represents a study–bacterium combination; bubble colour denotes direction of change (increase, no change, decrease), and bubble size indicates the number of studies reporting that pattern for a given taxon. All observations except those from Wang et al. [40] derive from targeted analyses of pre-specified taxa; the figure therefore summarises change in selected taxa and does not represent community-level (microbiome) change.

**Table 1:** Characteristics of included studies.

Author, Year, Country and Study Design	Participant Characteristics and Periodontal Disease Status	NSPT Delivery and Adjunctive Treatments	Time Intervals	Microbiota Sampling Sites	Measured Clinical Outcomes	Risk of Bias Tools Used and Assessment Score *
Almeida et al., 2019, Brazil [27], RCT	54 participants ≥ 35 years old 28 non-diabetic + PD and 26 T2DM + PD Mild to moderate PD	FMSRP + 0.12% CHX solution (1 min) + pockets irrigated with 1% CHX gel (3×). Dorsum of the tongue brushed with same gel for 1 min. OHI using 0.12% CHX	Baseline, 3 months, 6 months, 9 months, 12 months	Saliva	PPD, CAL, BOP, plaque accumulation	RoB 2 Score: Some concerns
Buzinin et al., 2014, Malaysia [28], interventional controlled clinical trial	41 participants between 20 and 65 years old 21 non-diabetic + PD and 20 T1DM + PD Moderate to advanced PD	FMD in a single session using Gracey curette+ ultrasonic scaling	Baseline, 2 months, 3 months	Subgingival biofilm from the deepest pocket of each quadrant	PPD, CAL, BOP, plaque score	ROBINS I Score: Moderate risk
Christgau et al., 1998, Germany [29], prospective parallel study	40 participants, >30 years old (16F, 24M) 20 non-diabetic + PD and 20 T1DM/T2DM + PD Moderate to advanced PD	Patient motivation, OHI, SRP, removal of overhanging cervical crown margins, extracting hopeless teeth, and splinting mobile teeth NSPT: subgingival SRP with hand instruments under LA and with one session per quadrant. Pockets irrigated with a 0.2% CHX solution + 1% CHX gel	Baseline, 2 weeks, 4 months	Subgingival biofilm from the deepest pocket in each quadrant	PPD, CAL, PI	ROBINS I Score: Moderate risk
da Cruz et al., 2008, Brazil [30], interventional study	20 participants between 30 and 70 years old 10 non-diabetic + PD and 10 T2DM + PD Generalised chronic PD	FMSRP in all diseased sites under LA in one session ~2 h. OHIs (tooth-brushing technique, interdental cleaning, and use of tongue scrapers) were given	Baseline, 3 months	Subgingival biofilm from the deepest pocket	PPD, CAL	RoB 2 Score: Some concerns
Devina et al., 2024, Indonesia [31], parallel, double-blind RCT	38 participants between 40 and 65 years old 18 non-diabetic + PD and 18 T2DM + PD PD as classified by EFP 2017	FMSRP + OHI The test and control groups were randomly assigned to receive a placebo gel (control group) or 0.2% HA (hyaluronic acid) gel (test group)	Baseline, 4 weeks	Subgingival plaque	PPD, CAL, BOP	RoB 2 Score: Some concerns
Duarte et al., 2018, Brazil [32], prospective cohort study	58 participants 29 non-diabetic + PD and 29 T2DM + PD Severe PD according to Armitage, 1999	SRP combined with metronidazole (400 mg thrice a day) and amoxicillin (500 mg thrice a day) for 14 days	Baseline, 3 months, 6 months, 12 months	Subgingival biofilm	PPD, CAL, BOP, plaque accumulation	RoB 2 Score: Low risk
Hropot Plesko et al., 2021, Slovenia [33], interventional controlled clinical trial	33 participants (19F, 14M) 11 non-diabetic and PD and 11 T1DM and PD and 11 T2DM and PD All patients diagnosed with at least stage II PD, grade B or C (chronic PD)	NSPT with full-mouth disinfection protocol. Supragingival plaque removal + SRP. Tongue scraped + polished + pockets rinsed with 0.12% CHX (2 min). OHI instruction (rinsing the mouth 2×/day for 2 min with 0.12% CHX for 2 weeks)	Baseline, 3 months	Plaque samples from the two deepest pockets	PPD, CAL, BOP, plaque score (full mouth)	ROBINS I Score: Moderate risk
Lakhani et al., 2019, India [34], interventional cohort study	120 participants 30 non-diabetic + PD and 90 T2DM + PD split into 30 with good control (HbA1c < 5%), 30 with moderate control (HbA1c < 7%) and 30 with poor control (HbA1c > 8%) Mild to moderate PD	All patients were subjected to ultrasonic SRP	Baseline, 1 month, 4 months	Subgingival plaque samples were collected from the deepest part of the pocket	PPD, relative attachment level, gingival index, PI	ROBINS I Score: Serious risk
Mirnić et al., 2021, Serbia [35], prospective interventional study	92 participants between 30 and 70 years old 31 non-diabetic + PD and 61 T2DM + PD separated into well-controlled DM and poorly controlled DM Chronic PD	NSPT comprising SRP (ultrasonic device + Gracey curettes) and OHI for home care	Baseline, 3 months	Subgingival plaque	PPD CAL, PI	ROBINS I Score: Low risk
Rajpurohit et al., 2024, India [36], prospective observational cohort study	45 participants all over 30 years old 15 without PD and 15 non-diabetic + PD and 15 T2DM + PD Chronic PD	SRP	Baseline, 6 weeks	Subgingival		ROBINS I Score: Moderate risk
Rode et al., 2021, India [37], interventional cohort study	60 participants between 35 and 65 years old 20 non-diabetic + PD and 20 T2DM + PD and 20 without PD Moderate to severe PD	NSPT	Baseline, 1 month, 3 months	Saliva	PPD, CAL	ROBINS I Score: Serious risk
Sambashivaiah et al., 2011, India [38], RCT	36 participants (25M and 11M) 12 without PD and 12 non-diabetic + PD and 12 T2DM + PD Chronic PD: PPD > 5 mm	FMSRP under local anaesthesia and instruction in proper home care procedure	Baseline, 2 weeks	Subgingival plaque from the deepest pocket		RoB 2 Score: Some concerns
Silva-Boghossian et al., 2014, Brazil [39], controlled clinical trial	40 participants (22F, 18M) 20 non-diabetic + PD and 20 T2DM + PD Moderate to severe PD	OHI and four weekly sessions of SRP (1 h each) under local anaesthesia, and professional plaque control twice a month for 3 months	Baseline, 3 months	Subgingival biofilm	PPD, CAL, BOP	RoB 2 Score: Some concerns
Wang et al., 2023, China [40], prospective parallel study	22 participants. 11 non-diabetic + PD and 11 T2DM + PD Chronic PD	Patients were instructed to use the modified Bass technique and were treated with NSPT consisting of SRP. At the 3-month visit, participants were given OHI again	Baseline, 3 months	Subgingival plaque, saliva	PPD, alpha diversity, CAL, PI	ROBINS I Score: Serious risk

BOP, bleeding on probing; CAL, clinical attachment loss/level; CHX, chlorhexidine; DM, diabetes mellitus; EFP, European Federation of Periodontology; F, female; FMD, full-mouth debridement; FMSRP, full-mouth scaling and root planing; HA, hyaluronic acid; LA, local anaesthesia; M, male; ND, non-diabetic; NSPT, non-surgical periodontal therapy; OHI, oral hygiene instruction; PCR, polymerase chain reaction; PD, periodontitis; PI, plaque index; PPD, periodontal probing depth; RCT, randomised controlled trial; SRP, scaling and root planing; T1DM, type 1 diabetes mellitus; T2DM, type 2 diabetes mellitus; 16S rRNA, 16S ribosomal RNA gene sequencing. Armitage (1999) classification system for periodontal diseases, which is defined as slight/mild (1–2 mm clinical attachment loss, CAL), moderate (3–4 mm CAL) or severe (≥5 mm CAL). * Studies were assessed using the revised Cochrane risk-of-bias tool for randomised trials (RoB 2) or the Risk of Bias in Non-randomised Studies of Interventions (ROBINS-I) tool. Risk of bias scores range from low risk to serious risk (ROBINS-I) or low risk/some concerns/high risk (RoB 2).

**Table 2:** Microbiological outcomes following non-surgical periodontal therapy in diabetic and non-diabetic adults with periodontitis.

Author, Year	Bacterial Analysis Method and Taxa/Bacterial Complex	Baseline	Outcome ^†^	Key Between-Group Finding	Overall Summary
Almeida et al., 2019 [27]	PCR *T. denticola*, *P. gingivalis*, *T. forsythia* (Red complex)	Non-diabetic + PD: PG: 2.51 ± 1.62, TD: 8.23 ± 10.96, TF: 4.71 ± 1.30 Diabetic + PD: PG: 1.62 ± 1.86, TD: 4.35 ± 5.19, TF: 3.69 ± 1.80.	6 months Non-diabetic + PD: PG: 1.93 ± 1.92, TD: 7.23 ± 9.36, TF: **4.48 ± 0.97** Diabetic + PD: PG: 0.94 ± 1.35, TD: 2.59 ± 3.05, TF: **3.37 ± 2.23** 12 months Non-diabetic + PD: PG: 2.83 ± 2.19, TD: 9.31 ± 9.81, TF: 4.95 ± 0.79 Diabetic + PD: PG: 2.16 ± 2.37, TD: 3.76 ± 5.41, TF: 3.72 ± 1.32	TF significantly lower in diabetics vs. ND at 6 months	FMD did not promote changes in the salivary levels of pathogens in either of the groups except for significant decrease in the mean levels of *T. forsythia* compared to baseline for the diabetic group at 6 months post-NSPT compared to non-diabetic group.
Buzinin et al., 2014 [28]	PCR *P. gingivalis*, *T. forsythia*, *A. actinomycetemcomitans*	Non-diabetic: PG: 1.8 × 10^7^ ± 2.6 × 10^7^, TF: 3.0 × 10^4^ ± 2.4 × 10^4^, AA: 4.1 × 10^3^ ± 8.5 × 10^3^ Diabetic: PG: 1.4 × 10^7^ ± 2.3 × 10^7^, F: 2.7 × 10^4^ ± 2.0 × 10^4^, AA: 7.0 × 10^3^ ± 1.2 × 10^4^	3 months Non-diabetic: PG: 3.4 × 10^6^ ± 9.5 × 10^6^, TF: 8.2 × 10^3^ ± 1.6 × 10^4^, AA: 6.6 × 10^3^ ± 1.4 × 10^4^ Diabetic: PG: **7.2 × 10^4^ ± 2.7 × 10^4^**, TF: **5.2 × 10^3^ ± 1.1 × 10^4^**, AA: 1.3 × 10^3^ ± 4.1 × 10^3^	Larger PG and TF decrease in diabetics than ND	There was a significant decrease in the quantity of *P. gingivalis* and *T. forsythia* compared to baseline for both the diabetic and ND group at 3 months post NSPT.
Christgau et al., 1998 [29]	Agar *P. gingivalis*, *F. nucleatum*, *A. actinomycetemcomitans*, *E. corrodens*, *P.intermedia*, *P. micros*, *Veillonella* species	Non-diabetic: AA: 8 (50%), PG: 16 (120%), PI: 15 (115%), FN: 16 (125%), EC: 0, PM: 0, VSP: 13 (95%) Diabetic: AA: 6 (37.5%), PG: 15 (102%), PI: 15 (110%), FN: 14 (100%), EC: 0, PM: 1 (7.5%), VSP 10 (60%)	2 weeks Non-diabetic: AA: 5 (30%), PG: 18 (140%), PI: 13 (105%), FN: 19 (175%), EC: 1 (5%), PM: 0, VSP: 11 (75%) Diabetic: AA: 6 (30%), PG: 17 (120%), PI: 12 (80%), FN: 17 (125%), EC: 0, PM: 0, VSP: 11 (75%) 4 months Non-diabetic: AA: 4 (20%), PG: 8 (65%), PI: 16 (95%), FN: 17 (125%), EC: 1 (5%), PM: 0, VSP: 8 (60%) Diabetic: AA: 4 (22.5%), PG: 12 (75%), PI: 13 (90%), FN: 16 (120%), EC: 0, PM: 0, VSP: 6 (40%)	No consistent difference between ND and DM	Within the non-diabetic group and diabetic group, there was a decrease in *A. actinomycetemcomitans*, *P. gingivalis* and *Veillonella* species from baseline to 4 months.
da Cruz et al., 2008 [30] ^‡^	PCR *P. gingivalis*, *T. forsythia*, *A. actinomycetemcomitans*	Non-diabetic with PD: AA: 50%, PG: 80%, TF: 100% Diabetic with PD: AA: 40%, PG: 80%, TF: 50%	3 months Non-diabetic + PD: AA: 50%, PG: 70%, TF: 50% Diabetic + PD: AA: 40%, PG: 60%, TF: 30%	No clear difference between ND and DM	There was a decrease in the prevalence of *P. gingivalis* and *T. forsythia* in both the non-diabetic and diabetic groups from baseline to 3 months.
The percentages of sites with furcation					
The percentages of sites with PPD ≥ 5 mm		Non-diabetic + PD: AA: 20%, PG: 80%, TF: 100% Diabetic with PD: AA: 20%, PG: 80%, TF: 40%	3 months Non-diabetic + PD: AA: 10%, PG: 70%, TF: 40% Diabetic + PD: AA: 20%, PG: 70%, TF: 40%		There was a decrease in the prevalence of *A. actinomycetemcomitans*, *P. gingivalis* and *T. forsythia* in the non-diabetic and diabetic group.
Devina et al., 2024 [31]	PCR *P. gingivalis*, *F. nucleatum*	Non-diabetic (placebo gel): PG: 1.00 ± 0.78, FN: 1.00 ± 0.81 Non-diabetic (hyaluronic acid gel): PG: 1.00 ± 1.55, FN: 1.00 ± 0.47 Diabetic (placebo gel): PG: 1.00 ± 1.45, FN: 1.00 ± 1.31 Diabetic: (hyaluronic acid gel): PG: 1.00 ± 1.63, FN: 1.00 ± 0.70	4 weeks Non-diabetic (placebo gel): PG: **0.20 ± 0.2**, FN: 0.93 ± 1.00 Non-diabetic (hyaluronic acid gel): PG: 0.42 ± 0.54, FN: 0.76 ± 0.59 Diabetic (placebo gel): PG: **0.50 ± 1.17**, FN: 0.76 ± 0.96 Diabetic: (hyaluronic acid gel): PG: 2.07 ± 2.81, FN: 0.99 ± 1.33	Placebo produced larger PG reductions than hyaluronic acid gel in both ND and DM	The relative microbial plaque expression for *P. gingivalis* from baseline to 4 weeks was significantly decreased for both the non-diabetic and diabetic groups using the placebo gel.
Duarte et al., 2018 [32]	Checkerboard DNA-DNA hybridization 40 bacterial species	Non-diabetic with PD: Red complex: 24.7 Orange complex: 28.7 Green complex: 4.4 Actinomyces species: 10.4 Diabetic with PD: Red complex: 17.8 Orange complex: 24.1 Green complex: 5.8 Actinomyces species: 29.7	12 months Non-diabetic + PD: **red complex: 3.2**, orange complex: 18, **green complex: 11**, **Actinomyces species: 33.2** Diabetic + PD: **red complex: 5.7**, orange complex: 23.4, **green complex: 9.1**, Actinomyces species: 34.9	Similar directional shifts in ND and DM	There was a significant decrease in the number of red and green complex bacteria from baseline to 12 months. Diabetic group had higher mean proportions of the red and orange complexes than the non-diabetic group.
Hropot Plesko et al., 2021 [33]	PCR *P. gingivalis*, *T. forsythia*, *A. actinomycetemcomitans*, *T. denticola*, *P. intermedia*	Non-diabetic and PD: AA: 18.1 ± 4.0, PG: 68.1 ± 5.0, PI: 59.1 ± 5.0, TD: 91.0 ± 2.9, TF: 100.0 ± 0 T1DM + PD: AA: 18.1 ± 4.0, PG: 27.2 ± 4.6, PI: 41.0 ± 5.0, TD: 81.8 ± 3.9, TF: 86.4 ± 3.5 T2DM and PD: AA: 22.7 ± 4.0, PG: 72.7 ± 4.6, PI: 45.5 ± 5.1, TD: 82.0 ± 3.9, TF: 100.0 ± 0	3 months Non-diabetic and PD: AA: 13.6 ± 4.0, PG: **59.1 ± 5.0**, Pi: 40.9 ± 5.0, TD: 81.8 ± 3.9, TF: 91.0 ± 2.9 T1DM and PD: **AA: 40.9 ± 5.0**, PG: 27.3 ± 4.6, PI: 41.0 ± 5.0, TD: 73.0 ± 4.6, TF: 72.7 ± 4.6 T2DM and PD: AA: 18.1 ± 4.0, PG: 68.2 ± 4.8, PI: 50.0 ± 5.1, TD: 77.2 ± 4.3, TF: 100 ± 0	Strongest PG fall in ND; AA change most marked in T1DM	There was a significant decrease for the non-diabetic group in the prevalence (%) of *P. gingivalis* from baseline to 3 months. There was a significant decrease for the Type 1 diabetic group in the prevalence (%) of *A. actinomycetemcomitans* from baseline to 3 months.
Lakhani et al. 2019 [34]	PCR *P. gingivalis*, *T. forsythia*, *P. Intermedia*	Only mean differences reported; raw counts not available	Only mean differences reported; raw counts not available	No significant between-group differences	Significant within-group reductions in *P. gingivalis*, *T. forsythia* and *P. intermedia* in both diabetic and non-diabetic groups.
Mirnić et al., 2021 [35]	PCR *P. gingivalis*, *T. forsythia*, *P. Intermedia*	Non-diabetic with PD: Aa: 22 (71.0%), PG: 27 (87.1%), PI: 24 (77.4%), TF: 25 (80.6%) Well-controlled diabetes: Aa: 14 (48.3%) PG: 21 (72.4%) PI: 25 (86.2%) TF: 28 (96.6%) Poorly controlled diabetes: Aa: 14 (43.8%), PG: 20 (62.5%), Pi: 24 (75.0%), TF: 26 (81.3%)	3 months Non-diabetic with PD: **AA: 11 (35.5%)**, **PG: 17 (54.8%)**, PI: 19 (61.3%), **TF: 19 (61.3%)** Well-controlled diabetes: AA: 9 (31.0%), **PG: 15 (51.7%)**, PI: 21 (72.4%), TF: 23 (79.3%) Poorly controlled diabetes: **AA: 4 (12.5%)**, PG: 15 (46.9%), **PI: 14 (43.8%)**, **TF: 18 (56.3%)**	Poorly controlled T2DM showed marked drops in AA, PI, TF	From baseline, there was a significant decrease in the percentage of patients with *A. actinomycetemcomitans*, *P. gingivalis* and *T. forsythia* for the non-diabetic group, *P. gingivalis* for the well-controlled DM group, and *A. actinomycetemcomitans*, *P. intermedia* and *T. forsythia* for the poorly controlled DM group.
Rajpurohit et al., 2024 [36]	PCR *Mogibacterium timidum*	Non-diabetic+ non-PD: 5.31× 10^5^ ± 16.69 × 10^5^ Non-diabetic + PD: 409.56 × 10^5^ ± 762.94 × 10^5^ Diabetes + PD: 50,828.33 × 10^5^ ± 101,099.0 × 10^5^	6 weeks Non-diabetic + non-PD: **2.46 × 10^5^ ± 3.74 × 10^5^** Non-diabetic + PD: **45.95 × 10^5^ ± 110.85 ×10^5^** Diabetes + PD: **237.96 × 10^5^ ± 482.07 × 10^5^**	Diabetics retained higher *M. timidum* load post-treatment	There was a significant decrease in the counts of *M. timidum* for all three groups from baseline to 6 weeks.
Rode et al., 2021 [37]	PCR *P. gingivalis*	Non-PD: 0 Non-diabetic and PD: 19 T2DM and PD: 20	4 weeks Non-PD: 0 Non-diabetic and PD: 3 T2DM and PD: 5 3 months Non-PD: 0 Non-diabetic and PD: 1 T2DM and PD: 3	Reductions similar in ND and diabetic periodontitis	There was a decrease among the patients presenting with *P. gingivalis* for both diabetic and non-diabetic groups from baseline to 3 months.
Sambashivaiah et al., 2011 [38]	Rapid urease test *Helicobacter pylori*	Non-diabetic with PD: 75% Diabetic with PD: 83.3%	2 weeks **Non-diabetic with PD: 0%** **Diabetic with PD: 8.30%**	Similar reductions in ND and diabetics	There was a significant decrease in the incidence of *H. Pylori* in both diabetic and non-diabetic groups from baseline to 2 weeks.
Silva-Boghossian et al., 2014 [39]	Checkerboard DNA-DNA hybridization Species richness, microbial diversity of 45 bacterial species	Non-diabetic with PD: *Streptococcus* spp., *F. nucleatum*, *P. intermedia* and red-complex species (*P. gingivalis*, *T. denticola*, *T. forsythia*) Diabetic with PD: *P. gingivalis*, *T. forsythia*, *T. denticola*, *F. nucleatum subspecies*, *C. rectus* and *E. nodatum*	Red and orange complex taxa such as *P. gingivalis*, *T. forsythia*, *T. denticola*, *P. intermedia* and *F. nucleatum* subspecies showed reduction over time for both the groups. Diabetic participants exhibited higher prevalence of *Agerencseriae*, *A. naeslundii*, *A. oris*, *A. odontolyticus*, *C. sputigena*, *F. periodonticum*, and *G. morbillorum*) than non-diabetic individuals	NSPT reduced key pathogens in both diabetic and ND groups, with no consistent advantage for either group	Diabetic subjects showed increases in host-compatible taxa, particularly *Actinomyces* spp. and *G. morbillorum*.
Wang et al., 2023 [40]	16S rRNA Alpha diversity measured using observed features, Shannon and Simpson index beta diversity using UniFrac distances Relative abundance of top 10 species and differential abundance analysis	Diabetic with PD: higher proportion of *Porphyromonas*, *Fretibacterium*, *Fusobacterium*, *Rothia*, and *Filifactor*, *Eubacterium saphenum* in subgingival plaque	Diabetic with PD: NSPT increased subgingival microbial diversity and the relative abundance of health-associated bacteria while proportionally reducing periodontal pathogens. *Porphyromonas*, *Filifactor* and *Eubacterium* decreased, whereas the levels of health-associated *Selenomonas*, *Actinobacteria*, *Streptococcus*, *Veillonella*, *Actinomyces*, *Leptotrichia* and *Prevotella* increased.	Alpha and beta diversity showed no difference between the non-diabetic and diabetic group	Diabetic patients showed a distinct pattern of community restructuring characterised by increased diversity, reduced pathogen proportions and a denser, more stable co-occurrence network.

Bolded text represents significant results in the baseline and post-treatment change column as reported by the original studies (*p* < 0.05). ^†^ Unless otherwise specified, values are presented as bacterial counts (e.g., colony-forming units or genome copies), proportions, or percentages of positive sites or patients for each species. Where “red complex”, “orange complex”, “green complex”, and Actinomyces species are reported, the values represent the mean proportions of total bacterial DNA detected in subgingival samples. ^‡^ da Cruz et al. note: This study reported two related site-level outcomes: (1) percentage of furcation sites colonised by AA, PG and TF at baseline and 3 months after full-mouth SRP, and (2) percentage of sites with PD ≥ 5 mm colonised by these pathogens at baseline and 3 months. All studies except Wang et al. used targeted methods restricted to pre-specified taxa (culture, PCR, rapid urease test or checkerboard DNA–DNA hybridisation); only Wang et al. used 16S rRNA gene sequencing and provided community-level (microbiome) data. Species richness and diversity reported by Silva-Boghossian et al. were derived from a 45-species checkerboard panel and are not community-level measures. AA, *Aggregatibacter actinomycetemcomitans*; BOP, bleeding on probing; CAL, clinical attachment level; CP, chronic periodontitis; DM, diabetes mellitus; EC, *Eikenella corrodens*; FMD, full-mouth debridement; FN, *Fusobacterium nucleatum*; HbA1c, glycated haemoglobin A1c; MT, *Mogibacterium timidum*; ND, non-diabetic; NSPT, non-surgical periodontal therapy; PD, periodontitis; PG, *Porphyromonas gingivalis*; PI, *Prevotella intermedia*; PM, *Parvimonas micra*; SH, systemically healthy; SRP, scaling and root planing; T1DM, type 1 diabetes mellitus; T2DM, type 2 diabetes mellitus; TD, *Treponema denticola*; TF, *Tannerella forsythia*; VPI, visible plaque index; VSP, *Veillonella* species. PCR, polymerase chain reaction; 16S rRNA, 16S ribosomal RNA gene sequencing; checkerboard DNA–DNA hybridisation, membrane-based hybridisation assay for simultaneous detection of multiple bacterial species; agar, culture on selective/non-selective media.

## Data Availability

No new datasets were created or analysed in this study. Data sharing is not applicable to this article.

## Supplementary Materials

The following supporting information can be downloaded at https://www.mdpi.com/article/10.3390/dj14100617/s1: Table S1: PRISMA 2020 Checklist; Table S2: Inclusion and exclusion criteria; Table S3: Database and search strings; Table S4: Excluded studies and reasons for exclusion; Table S5: Diabetes characteristics and glycaemic control of participants in the included studies; Table S6: Periodontal diagnostic criteria, disease severity and treatment protocol of the included studies; Table S7: Summary of changes in periodontal probing depth at baseline and following non-surgical periodontal therapy; Table S8: Summary of changes in periodontal clinical attachment level at baseline and following non-surgical periodontal therapy; Table S9: Summary of changes in bleeding on probing following non-surgical periodontal therapy; Table S10: Summary of changes in plaque levels following non-surgical periodontal therapy; Table S11: GRADE summary of findings: non-surgical periodontal therapy in adults with periodontitis with and without diabetes; Figure S1: Cochrane risk-of-bias (RoB) tool for randomised trials (RoB 2) summary; Figure S2: Cochrane Risk of Bias in Non-randomised Studies of Interventions (ROBINS) tool summary.

## References

[1] Könönen E., Gursoy M., Gursoy U.K. (2019). Periodontitis: A Multifaceted Disease of Tooth-Supporting Tissues. J. Clin. Med..

[2] Nazir M.A. (2017). Prevalence of periodontal disease, its association with systemic diseases and prevention. Int. J. Health Sci..

[3] Yang Y., Sun X., Yang Y., Qie Y. (2024). Insight of the interrelationship and association mechanism between periodontitis and diabetes mellitus. Regen. Ther..

[4] Preshaw P.M., Bissett S.M. (2019). Periodontitis and diabetes. Br. Dent. J..

[5] Nath S., Weyrich L., Zilm P., Kapellas K., Jamieson L.M. (2024). Oral microbiome research from a public health perspective and implications for oral health. Community Dent. Health.

[6] Cui Z., Wang P., Gao W. (2025). Microbial dysbiosis in periodontitis and peri-implantitis: Pathogenesis, immune responses, and therapeutic. Front. Cell. Infect. Microbiol..

[7] Holt S.C., Ebersole J.L. (2005). Porphyromonas gingivalis, Treponema denticola, and Tannerella forsythia: The “red complex”, a prototype polybacterial pathogenic consortium in periodontitis. Periodontology 2000.

[8] Nath S., Pulikkotil S.J., Weyrich L., Zilm P., Kapellas K., Jamieson L. (2022). Effect of Periodontal Interventions on Characteristics of the Periodontal Microbial Profile: A Systematic Review and Meta-Analysis. Microorganisms.

[9] Ganesan S.M., Joshi V., Fellows M., Dabdoub S.M., Nagaraja H.N., O’Donnell B., Deshpande N.R., Kumar P.S. (2017). A tale of two risks: Smoking, diabetes and the subgingival microbiome. ISME J..

[10] Page R.C., Offenbacher S., Schroeder H.E., Seymour G.J., Kornman K.S. (1997). Advances in the pathogenesis of periodontitis: Summary of developments, clinical implications and future directions. Periodontology 2000.

[11] Van Dyke T.E., Sima C. (2020). Understanding resolution of inflammation in periodontal diseases: Is chronic inflammatory periodontitis a failure to resolve?. Periodontology 2000.

[12] Tonetti M.S., Greenwell H., Kornman K.S. (2018). Staging and grading of periodontitis: Framework and proposal of a new classification and case definition. J. Periodontol..

[13] Simpson T.C., Clarkson J.E., Worthington H.V., MacDonald L., Weldon J.C., Needleman I., Iheozor-Ejiofor Z., Wild S.H., Qureshi A., Walker A. (2022). Treatment of periodontitis for glycaemic control in people with diabetes mellitus. Cochrane Database Syst. Rev..

[14] Qin H., Li G., Xu X., Zhang C., Zhong W., Xu S., Yin Y., Song J. (2022). The role of oral microbiome in periodontitis under diabetes mellitus. J. Oral Microbiol..

[15] Chen X., Jamieson L., Weyrich L.S., Nath S. (2026). Global Landscape of Publicly Available Human Oral Microbiome Data. J. Dent. Res..

[16] Baeza M., Morales A., Cisterna C., Cavalla F., Jara G., Isamitt Y., Pino P., Gamonal J. (2019). Effect of periodontal treatment in patients with periodontitis and diabetes: Systematic review and meta-analysis. J. Appl. Oral Sci..

[17] Teshome A., Yitayeh A. (2016). The effect of periodontal therapy on glycemic control and fasting plasma glucose level in type 2 diabetic patients: Systematic review and meta-analysis. BMC Oral Health.

[18] Lalla E., Cheng B., Lal S., Kaplan S., Softness B., Greenberg E., Goland R.S., Lamster I.B. (2007). Diabetes mellitus promotes periodontal destruction in children. J. Clin. Periodontol..

[19] Listgarten M.A. (1980). Periodontal probing: What does it mean?. J. Clin. Periodontol..

[20] Tugnait A., Clerehugh V., Hirschmann P.N. (2000). The usefulness of radiographs in diagnosis and management of periodontal diseases: A review. J. Dent..

[21] Newbrun E. (1996). Indices to measure gingival bleeding. J. Periodontol..

[22] Silness J., Loe H. (1964). Periodontal Disease in Pregnancy. II. Correlation Between Oral Hygiene and Periodontal Condtion. Acta Odontol. Scand..

[23] Sterne J.A., Savović J., Page M.J., Elbers R.G., Blencowe N.S., Boutron I., Cates C.J., Cheng H.Y., Corbett M.S., Eldridge S.M. (2019). RoB 2: A revised tool for assessing risk of bias in randomised trials. BMJ.

[24] Sterne J.A.C., Hernán M.A., Reeves B.C., Savović J., Berkman N.D., Viswanathan M., Henry D., Altman D.G., Ansari M.T., Boutron I. (2016). ROBINS-I: A tool for assessing risk of bias in non-randomized studies of interventions. BMJ.

[25] Prasad M. (2024). Introduction to the GRADE tool for rating certainty in evidence and recommendations. Clin. Epidemiol. Glob. Health.

[26] Song W.J., Kang W.Y., Liu X.M., Sun L., Feng Q., Ge S.H. (2022). Study on the dynamic changes of oral microbiota in type 2 diabetes patients with periodontitis after initial periodontal therapy. Chin. J. Stomatol..

[27] Almeida M.L., Duarte P.M., Figueira E.A., Lemos J.C., Nobre C.M.G., Miranda T.S., de Vasconcelos Gurgel B.C. (2020). Effects of a full-mouth disinfection protocol on the treatment of type-2 diabetic and non-diabetic subjects with mild-to-moderate periodontitis: One-year clinical outcomes. Clin. Oral Investig..

[28] Buzinin S.M., Alabsi A.M., Tan A.T.B., Vincent-Chong V.K. (2014). Swaminathan Effects of Nonsurgical Periodontal Therapy on Clinical Response, Microbiological Profile, and Glycemic Control in Malaysian Subjects with Type 1 Diabetes. Sci. World J..

[29] Christgau M., Palitzsch K., Schmalz G., Kreiner U., Frenzel S. (2005). Healing response to non-surgical periodontal therapy in patients with diabetes mellitus: Clinical, microbiological, and immunologic results. J. Clin. Periodontol..

[30] da Cruz G.A., de Toledo S., Sallum E.A., Sallum A.W., Ambrosano G.M.B., Sardi J.d.C.O., da Cruz S.E.B., Gonçalves R.B. (2008). Clinical and Laboratory Evaluations of Non-Surgical Periodontal Treatment in Subjects with Diabetes Mellitus. J. Periodontol..

[31] Devina A.A., Halim F.C., Meivi M., Masulili S.L.C., Tadjoedin E.S.S., Lessang R., Widaryono A., Bachtiar B.M., Sulijaya B., Tadjoedin F.M. (2024). Effectiveness of 0.2% Hyaluronic Acid on Clinical, Biomolecular and Microbiological Parameters in Type 2 Diabetes Mellitus Patients with Periodontitis. Eur. J. Dent..

[32] Duarte P.M., Feres M., Yassine L.L.S., Soares G.M.S., Miranda T.S., Faveri M., Retamal-Valdes B., Figueiredo L.C. (2018). Clinical and microbiological effects of scaling and root planing, metronidazole and amoxicillin in the treatment of diabetic and non-diabetic subjects with periodontitis: A cohort study. J. Clin. Periodontol..

[33] Plesko N.H., Skaleric E., Seme K., Janez A., Skaleric U., Gaspirc B. (2021). Effect of Full-mouth Disinfection Protocol on Glycemic Control and Subgingival Microbiota in Patients with Type 1 and Type 2 Diabetes. Quintessence Publ. Dtschl..

[34] Lakhani A., Sharma E., Sachdeva S., Thind S., Chaudhary K., Kapila S. (2019). Compare and Correlate the Presence of Bacterial Pathogen Concentrations and Glycaemic Status in Type 2 Diabetic and Non-Diabetic Patients with Chronic Periodontitis Following Phase 1 Therapy. J. Evol. Med. Dent. Sci..

[35] Mirnić J., Đurić M., Nikolić N., Veljović T., Gušić I., Petrović Ð., Milašin J. (2021). Clinical and Microbiological Assessment of Non-Surgical Treatment of Chronic Periodontitis in Controlled and Uncontrolled Type 2 Diabetic Patients. Acta Clin. Croat..

[36] Rajpurohit K.S., Dodwad V.M., Pharne P.M., Gavali N.V., Bhosale N., Khadtare Y. (2024). Comparative Evaluation of Mogibacterium timidum in Diabetic Patients with Chronic Periodontitis, Nondiabetic Patients with Chronic Periodontitis, and Healthy Patients During Pretreatment and Posttreatment: A Real-Time PCR Study. Cureus.

[37] Rode P.A., Rode P.A., Kolte A.P., Purohit H.J., Swami R.K. (2021). Evaluation and association of periodontal status with levels of Porphyromonas gingivalis in chronic periodontitis with and without Type 2 diabetes mellitus following nonsurgical periodontal therapy using quantitative polymerase chain reaction. An interventional study. J. Indian Soc. Periodontol..

[38] Sambashivaiah S., Bilichodmath S., Nanjaiah N., Kulal R. (2011). Helicobacter Pylori in periodontal pockets of chronic periodontitis patients with and without type II diabetes mellitus: A randomized controlled trial. Microbiol. Res..

[39] Silva-Boghossian C.M., Orrico S.R., Goncalves D., Correa F.O., Colombo A.P. (2014). Microbiological changes after periodontal therapy in diabetic patients with inadequate metabolic control. Braz. Oral Res..

[40] Wang L., Gao Z., Zhao Z., Shen X., Feng J., Xiong J. (2023). Oral microbiota in periodontitis patients with and without type 2 diabetes mellitus and their shifts after the nonsurgical periodontal therapy. Heliyon.

[41] Cruz D.F., Duarte P.M., Figueiredo L.C., da Silva H.D.P., Retamal-Valdes B., Feres M., Miranda T.S. (2021). Metronidazole and amoxicillin for patients with periodontitis and diabetes mellitus: 5-year secondary analysis of a randomized controlled trial. J. Periodontol..

[42] Campbell M., McKenzie J.E., Sowden A., Katikireddi S.V., Brennan S.E., Ellis S., Hartmann-Boyce J., Ryan R., Shepperd S., Thomas J. (2020). Synthesis without meta-analysis (SWiM) in systematic reviews: Reporting guideline. BMJ.

[43] Sbordone L., Ramaglia L., Gulletta E., Iacono V. (1990). Recolonization of the subgingival microflora after scaling and root planing in human periodontitis. J. Periodontol..

[44] Acharya Y., Taneja K.K., Haldar J. (2023). Dual functional therapeutics: Mitigating bacterial infection and associated inflammation. RSC Med. Chem..

[45] Shi R., Jamieson L., Nath S. (2025). Does non-surgical periodontal therapy improve biomarkers associated with cardiovascular disease? An umbrella review. Clin. Oral Investig..

