# Supplementary material for: Does Diabetes Influence the Microbiological Response to Non-Surgical Periodontal Therapy? A Systematic Review
Source: Dent J (Basel). 2026 Sep 23;14(10):617. doi: 10.3390/dj14100617 (PMC13703331; doi:10.3390/dj14100617)

Supplementary Files

Title: Does Diabetes Influence the Microbiological Response to Non-Surgical Periodontal Therapy? A Systematic Review

Table of Contents

Supplementary Tables 3

Supplementary Table S1. PRISMA 2020 Checklist 3

Supplementary Table S2. Inclusion and Exclusion Criteria 8

Supplementary Table S3. Database and Search Strings 9

Supplementary Table S4. Excluded Studies and Reasons for Exclusion 14

Supplementary Table S5. Diabetes characteristics and glycaemic control of participants in the included studies 20

Table S6. Periodontal diagnostic criteria, disease severity and treatment protocol of the included studies 24

Table S7. Summary of changes in periodontal probing depth at baseline and following non-surgical periodontal therapy 33

Supplementary Table S8. Summary of changes in periodontal clinical attachment level at baseline and following non-surgical periodontal therapy 37

Supplementary Table S9. Summary of changes in bleeding on probing following non-surgical periodontal therapy 40

Supplementary Table S10. Summary of changes in plaque levels following non-surgical periodontal therapy 42

Supplementary Table S11. GRADE summary of findings: non-surgical periodontal therapy in adults with periodontitis with and without diabetes 44

Supplementary Figures 46

Supplementary Figure S1. Cochrane Risk of Bias (RoB) Tool for Randomised Trials (RoB 2) Summary 46

Supplementary Figure S2. Cochrane Risk of Bias in Non-randomised Studies – of Interventions (ROBINS) Tool Summary 47

Supplementary Tables

Supplementary Table S1. PRISMA 2020 Checklist

Section and Topic	Item #	Checklist item	Location where item is reported
**TITLE**			
Title	1	Identify the report as a systematic review.	Title, Page 1
**ABSTRACT**			
Abstract	2	See the PRISMA 2020 for Abstracts checklist.	Abstract, Page 1
**INTRODUCTION**			
Rationale	3	Describe the rationale for the review in the context of existing knowledge.	Abstract, Section 1, Page 1,2,3
Objectives	4	Provide an explicit statement of the objective(s) or question(s) the review addresses.	Section 1 Page 3
**METHODS**			
Eligibility criteria	5	Specify the inclusion and exclusion criteria for the review and how studies were grouped for the syntheses.	Section 2.1., 2.1.1., 2.1.2., 2.1.3., 2.1.4., 2.1.5 Page 3-5
Information sources	6	Specify all databases, registers, websites, organisations, reference lists and other sources searched or consulted to identify studies. Specify the date when each source was last searched or consulted.	Section 2.2. Page 5
Search strategy	7	Present the full search strategies for all databases, registers and websites, including any filters and limits used.	Supplementary Table S2
Selection process	8	Specify the methods used to decide whether a study met the inclusion criteria of the review, including how many reviewers screened each record and each report retrieved, whether they worked independently, and if applicable, details of automation tools used in the process.	Section 2.3. Page 5
Data collection process	9	Specify the methods used to collect data from reports, including how many reviewers collected data from each report, whether they worked independently, any processes for obtaining or confirming data from study investigators, and if applicable, details of automation tools used in the	Section 2.4. Page 5,6

		process.	
Data items	10a	List and define all outcomes for which data were sought. Specify whether all results that were compatible with each outcome domain in each study were sought (e.g. for all measures, time points, analyses), and if not, the methods used to decide which results to collect.	Section 2.6. Page 4, 5
	10b	List and define all other variables for which data were sought (e.g. participant and intervention characteristics, funding sources). Describe any assumptions made about any missing or unclear information.	Section 2.6. Page 4
Study risk of bias assessment	11	Specify the methods used to assess risk of bias in the included studies, including details of the tool(s) used, how many reviewers assessed each study and whether they worked independently, and if applicable, details of automation tools used in the process.	Section 2.5. Page 6
Effect measures	12	Specify for each outcome the effect measure(s) (e.g. risk ratio, mean difference) used in the synthesis or presentation of results.	Section 2.6. Page 6,7
Synthesis methods	13a	Describe the processes used to decide which studies were eligible for each synthesis (e.g. tabulating the study intervention characteristics and comparing against the planned groups for each synthesis (item #5)).	Section 2.6. Page 6,7
	13b	Describe any methods required to prepare the data for presentation or synthesis, such as handling of missing summary statistics, or data conversions.	Section 2.6. Page 6,7
	13c	Describe any methods used to tabulate or visually display results of individual studies and syntheses.	Section 2.6. Page 6,7
	13d	Describe any methods used to synthesize results and provide a rationale for the choice(s). If meta-analysis was performed, describe the model(s), method(s) to identify the presence and extent of statistical heterogeneity, and software package(s) used.	Section 2.6. Page 6,7
	13e	Describe any methods used to explore possible causes of heterogeneity among study results (e.g. subgroup analysis, meta-regression).	Section 2.6. Page 6,7
	13f	Describe any sensitivity analyses conducted to assess robustness of the	Sectoin 2.6. Page 6,7

		synthesized results.	
Reporting bias assessment	14	Describe any methods used to assess risk of bias due to missing results in a synthesis (arising from reporting biases).	Section 2.5. Page 6
Certainty assessment	15	Describe any methods used to assess certainty (or confidence) in the body of evidence for an outcome.	Section 2.5. Page 6
**RESULTS**			
Study selection	16a	Describe the results of the search and selection process, from the number of records identified in the search to the number of studies included in the review, ideally using a flow diagram.	Section 3.1. Page 7-9
	16b	Cite studies that might appear to meet the inclusion criteria, but which were excluded, and explain why they were excluded.	Section 3.2. Page 7,8
Study characteristics	17	Cite each included study and present its characteristics.	Section 3, Page 7,8
Risk of bias in studies	18	Present assessments of risk of bias for each included study.	Supplementary Figure S1., Supplementary Figure S2.
Results of individual studies	19	For all outcomes, present, for each study: (a) summary statistics for each group (where appropriate) and (b) an effect estimate and its precision (e.g. confidence/credible interval), ideally using structured tables or plots.	Microbiological outcomes: Table 2, pages 14-18, and Figure 2, page 20 (Section 3.3, pages 9-21).<br>Clinical outcomes: Section 3.4, page 21, with Supplementary Tables S7 (PPD), S8 (CAL), S9 (BOP) and S10 (plaque). Study-level detail in Table 1, pages 11-13.
Results of syntheses	20a	For each synthesis, briefly summarise the characteristics and risk of bias among contributing studies.	Section 3.2, Page 9, Table 1, Page 11-13. Risk of bias in Section 3.5 Risk of Bias and GRADE Assessment, page 22, with Supplementary Figures S1 and S2
	20b	Present results of all statistical syntheses conducted. If meta-analysis was	Not applicable. No meta-analysis was

		done, present for each the summary estimate and its precision (e.g. confidence/credible interval) and measures of statistical heterogeneity. If comparing groups, describe the direction of the effect.	performed.
	20c	Present results of all investigations of possible causes of heterogeneity among study results.	Section 3.3, page 9
	20d	Present results of all sensitivity analyses conducted to assess the robustness of the synthesized results.	Not applicable.
Reporting biases	21	Present assessments of risk of bias due to missing results (arising from reporting biases) for each synthesis assessed.	Not applicable.
Certainty of evidence	22	Present assessments of certainty (or confidence) in the body of evidence for each outcome assessed.	Page 22, and Supplementary Table S11
**DISCUSSION**			
Discussion	23a	Provide a general interpretation of the results in the context of other evidence.	Section 4, pages 22-23.
	23b	Discuss any limitations of the evidence included in the review.	Section 4, pages 22-24.
	23c	Discuss any limitations of the review processes used.	Section 4, pages 22-24.
	23d	Discuss implications of the results for practice, policy, and future research.	Section 4 D, pages 24-25; Section 5.1 Implications for Clinical Practice and Section 5.2 Implications for Research, page 26.
**OTHER INFORMATION**			
Registration and protocol	24a	Provide registration information for the review, including register name and registration number, or state that the review was not registered.	Section 2, page 3: registered with PROSPERO, registration ID CRD420251005668; also stated in the Abstract.
	24b	Indicate where the review protocol can be accessed, or state that a protocol was not prepared.	Section 2, page 3: the protocol is available via the PROSPERO record (CRD420251005668) at https://www.crd.york.ac.uk/prospero/

	24c	Describe and explain any amendments to information provided at registration or in the protocol.	No amendments were made to the registered protocol.
Support	25	Describe sources of financial or non-financial support for the review, and the role of the funders or sponsors in the review.	Funding statement, page 26 (No funding was received for this review); non-financial support acknowledged in Acknowledgments, page 26.
Competing interests	26	Declare any competing interests of review authors.	Conflicts of Interest statement, page 27 (The authors report no conflict of interest).
Availability of data, code and other materials	27	Report which of the following are publicly available and where they can be found: template data collection forms; data extracted from included studies; data used for all analyses; analytic code; any other materials used in the review.	Data Availability Statement, page 26; extracted data are presented in Table 1 (pages 11-13), Table 2 (pages 14-18) and Supplementary Tables S1-S10; the search strings are in Supplementary Table S2. The data extraction form template and the R code used for the bubble plots (Section 2.6, page 8) are available from the corresponding author on request

Supplementary Table S2. Inclusion and Exclusion Criteria

Criteria	Population	Intervention	Control	Outcome	Study Type
**Inclusion Criteria**	Patients with periodontitis and diabetes	Non-surgical periodontal therapy	Periodontitis patients without diabetes	Studies reporting quantitative data on the oral microbiome shift following periodontal treatment in Periodontitis patients with Diabetes Mellitus	Case Control<br>Prospective Cohort Studies<br>Randomised Clinical Trials<br>Cohort trials<br>Clinical Trials
**Exclusion Criteria**	Population with any other medical conditions	Any other intervention	Any other population	Studies not reporting quantitative data on the oral microbiome shift	Animal, cadaver, in vitro studies, review articles, letters to the editor/editors, books/chapters, conference abstracts, personal opinions, textbooks, case reports, clinical studies

Supplementary Table S3. Database and Search Strings

Database	Search String	Date Searched	Number of Results
PubMed	(((Debridement, periodontal [MeSH]) OR (Therapy, periodontitis [MeSH]) OR "Periodontitis/therapy"[MeSH] OR "Periodontal diseases/therapy" [MeSH] OR "Subgingival Curettage"[MeSH] OR "Dental Scaling"[MeSH] OR "Periodontal Debridement/methods"[MeSH] OR "Root Planing"[MeSH] OR "Periodontal therap*"[tiab] OR "Dental scaling"[tiab] OR "Subgingival debridement"[tiab] OR "Periodontal treatment*"[tiab] OR "Periodontitis"[tiab] OR "Periodontal Diseases"[MeSH] OR "Periodontitis" [MeSH])<br>AND<br>("periodontitis"[mh] OR "gingivitis"[mh] OR "periodontal disease"[tiab] OR periodont*[tiab])<br><br>AND<br><br>("diabetes"[tiab] OR "Diabetes mellitus"[mh] OR diabet*[tiab] OR type 2 diabetes mellitus[tiab])<br><br>AND<br><br>(microbiota[mh] OR microb*[tw] OR microflora [tw] OR microo*[tw] OR bacteria[mh] OR bact*[tw] OR pathogen*[tw] OR species[tw] OR strain*[tw]))	2nd March 2025	1,581
Scopus	TITLE-ABS-KEY("periodontal debridement" OR "periodontitis therapy" OR "Periodontitis/therapy" OR "Periodontal diseases/therapy" OR "Subgingival Curettage" OR "Dental Scaling" OR "Periodontal Debridement/methods" OR "Root Planing" OR "Periodontal therap*" OR "Dental scaling" OR "Subgingival debridement" OR "Periodontal treatment*" OR "Periodontitis" OR "Periodontal Diseases")<br><br>AND<br><br>TITLE-ABS-KEY("periodontitis" OR "gingivitis" OR "periodontal disease" OR periodont*)<br><br>AND<br><br>TITLE-ABS-KEY("diabetes" OR "Diabetes mellitus" OR diabet* OR "type 2 diabetes mellitus")	7th March 2025	2,590

	AND<br><br>TITLE-ABS-KEY(microbiota OR microb* OR microflora OR microo* OR bacteria OR bact* OR pathogen* OR species OR strain*)		
Embase	("periodontal debridement" OR "periodontitis therapy" OR "Periodontitis/therapy" OR "Periodontal diseases/therapy" OR "Subgingival Curettage" OR "Dental Scaling" OR "Periodontal Debridement/methods" OR "Root Planing" OR Periodontal therap* OR "Dental scaling" OR "Subgingival debridement" OR "Periodontal treatment*" OR "Periodontitis" OR "Periodontal Diseases" OR "Periodontitis").ti,ab<br><br>AND<br><br>(“periodontitis” OR “gingivitis” OR “periodontal disease” OR periodont*).ti,ab<br><br>AND<br><br>(“diabetes” OR “Diabetes mellitus” OR diabet* OR “type 2 diabetes mellitus”).ti,ab<br><br>AND<br><br>(microbiota OR microb* OR microflora OR microo* OR bacteria OR bact* OR pathogen* OR species OR strain*).ti,ab	7th March 2025	1,218
Ovid Emcare	(“periodontal debridement” OR “periodontitis therapy” OR "Periodontitis/therapy” OR "Periodontal diseases/therapy" OR "Subgingival Curettage" OR "Dental Scaling" OR "Periodontal Debridement/methods" OR "Root Planing" OR Periodontal therap* OR "Dental scaling" OR "Subgingival debridement" OR "Periodontal treatment*" OR "Periodontitis" OR "Periodontal Diseases" OR "Periodontitis").ti,ab<br><br>AND<br><br>(“periodontitis” OR “gingivitis” OR “periodontal disease” OR periodont*).ti,ab	3rd March 2025	506

	AND<br><br>(“diabetes” OR “Diabetes mellitus” OR diabet* OR “type 2 diabetes mellitus”).ti,ab<br><br>AND<br><br>(microbiota OR microb* OR microflora OR microo* OR bacteria OR bact* OR pathogen* OR species OR strain*).ti,ab		
JBI EBP	(“periodontal debridement” OR “periodontitis therapy” OR "Periodontitis/therapy” OR "Periodontal diseases/therapy" OR "Subgingival Curettage" OR "Dental Scaling" OR "Periodontal Debridement/methods" OR "Root Planing" OR Periodontal therap* OR "Dental scaling" OR "Subgingival debridement" OR "Periodontal treatment*" OR "Periodontitis" OR "Periodontal Diseases" OR "Periodontitis").ti,ab<br><br>AND<br><br>(“periodontitis” OR “gingivitis” OR “periodontal disease” OR periodont*).ti,ab<br><br>AND<br><br>(“diabetes” OR “Diabetes mellitus” OR diabet* OR “type 2 diabetes mellitus”).ti,ab<br><br>AND<br><br>(microbiota OR microb* OR microflora OR microo* OR bacteria OR bact* OR pathogen* OR species OR strain*).ti,ab	3rd March 2025	0
Web of Science	TS=("periodontal debridement" OR "periodontitis therapy" OR "Periodontitis/therapy" OR "Periodontal diseases/therapy" OR "Subgingival Curettage" OR "Dental Scaling" OR "Periodontal Debridement/methods" OR "Root Planing" OR Periodontal therap* OR "Dental scaling" OR	7th March 2025	1,511

	"Subgingival debridement" OR "Periodontal treatment*" OR "Periodontitis" OR "Periodontal Diseases")<br>AND<br>TS=("periodontitis" OR "gingivitis" OR "periodontal disease" OR periodont*)<br>AND<br>TS=("diabetes" OR "Diabetes mellitus" OR diabet* OR "type 2 diabetes mellitus")<br>AND<br>TS=(microbiota OR microb* OR microflora OR microo* OR bacteria OR bact* OR pathogen* OR species OR strain*)<br><br>TS=("periodontal debridement" OR "periodontitis therapy" OR "Periodontitis/therapy" OR "Periodontal diseases/therapy" OR "Subgingival Curettage" OR "Dental Scaling" OR "Periodontal Debridement/methods" OR "Root Planing" OR "Periodontal therap*" OR "Dental scaling" OR "Subgingival debridement" OR "Periodontal treatment*" OR "Periodontitis" OR "Periodontal Diseases")<br>AND TS=("periodontitis" OR "gingivitis" OR "periodontal disease" OR periodont*)<br>AND TS=("diabetes" OR "Diabetes mellitus" OR diabet* OR "type 2 diabetes mellitus") AND TS=(microbiota OR microb* OR microflora OR microo* OR bacteria OR bact* OR pathogen* OR species OR strain*)		
Dentistry and Oral Sciences Source (DOSS)	DE "Periodontitis therapy" OR DE "Periodontal disease " OR DE "Periodontal therapy" OR TI "Subgingival Curettage" OR AB "Subgingival Curettage" OR DE "Dental Scaling" OR DE "periodontal debridement" OR DE "periodontitis therapy" OR DE "Periodontal Debridement" OR TI "Periodontal methods" OR AB "Periodontal methods" OR DE "Root Planing" OR TI "Periodontal therap*" OR AB "Periodontal therap*" OR TI "Subgingival debridement" OR AB "Subgingival debridement" OR DE "Periodontal treatment*" OR TI "Periodontitis" OR AB "Periodontitis" OR TI "Periodontal Diseases" OR AB "Periodontal Diseases" OR DE "Periodontitis"<br><br>AND<br><br>DE "Periodontitis" OR DE "Gingivitis" OR TI "Periodontal disease" OR AB "Periodontal disease" OR TI Periodont* OR AB Periodont*	3rd March 2025	515

	AND<br><br>TI “Diabetes” OR AB “Diabetes” OR DE “Diabetes mellitus” OR TI Diabet* OR AB Diabet* OR TI “type 2 diabetes mellitus” OR AB “type 2 diabetes mellitus”<br><br>AND<br><br>TI Microflora OR AB Microflora OR DE Microbiota OR TI microb* OR AB microb* OR TI microo* OR AB microo* OR DE bacteria OR TI bact* OR AB bact* OR TI pathogen* OR AB pathogen* OR TI species OR AB species OR TI strain* OR AB strain*		
Google Scholar	("periodontal debridement" OR "periodontitis therapy" OR "Periodontitis/therapy" OR "Periodontal diseases/therapy" OR "Subgingival Curettage" OR "Dental Scaling" OR "Periodontal Debridement/methods" OR "Root Planing" OR "Periodontal therap*" OR "Dental scaling" OR "Subgingival debridement" OR "Periodontal treatment*" OR "Periodontitis" OR "Periodontal Diseases")<br><br>AND (“periodontitis” OR “gingivitis” OR “periodontal disease” OR periodont*)<br><br>AND (“diabetes” OR “Diabetes mellitus” OR diabet* OR “type 2 diabetes mellitus”)<br><br>AND (microbiota OR microb* OR microflora OR microo* OR bacteria OR bact* OR pathogen* OR species OR strain*)	3rd March 2025	34

Supplementary Table S4. Excluded Studies and Reasons for Exclusion

Excluded Study	Reason for Exclusion
Metronidazole and amoxicillin for patients with periodontitis and diabetes mellitus: 5-year secondary analysis of a randomized controlled trial<br>Cruz, D. F. D.; Duarte, P. M.; Figueiredo, L. C.; da Silva, H. D. P.; Retamal-Valdes, B.; Feres, M.; Miranda, T. S.<br>Journal of Periodontology 2021	Wrong study type<br>Reviews patients of a previous randomised controlled trial - which did not have a control group (non-diabetic population)
Effect of Er: YAG laser on the dynamic changes in subgingival flora in type 2 diabetic patients with periodontitis<br>Guo, Bin; Liang, Qianqian; Lü, Yan; Fu, Min; Chen, Yuanyuan; Yuan, Yifang; Li, Yingchao<br>West China Journal of Stomatology 2014	Duplicate study<br>Study has been included in full text screening twice
Effect of Er: YAG laser on the dynamic changes in subgingival flora in type 2 diabetic patients with periodontitis<br>Guo, Bin; Liang, Qianqian; Lü, Yan; Fu, Min; Chen, Yuanyuan; Yuan, Yifang; Li, Yingchao<br>West China Journal of Stomatology 2014	Wrong intervention<br>No treatment for non-diabetic participants
Efficacy of non-surgical treatment accompanied by professional toothbrushing in the treatment of chronic periodontitis in patients with type 2 diabetes mellitus: a randomized controlled clinical trial<br>Lee, J. Y.; Choi, Y. Y.; Choi, Y.; Jin, B. H.<br>Journal of Periodontal and Implant Science 2020	Wrong outcome<br>Does not look at microbiological outcomes
Effect of diode laser application as an adjunct to nonsurgical periodontal therapy on the reduction of red complex microorganisms in type 2 diabetics with chronic periodontitis<br>Kocak, E.; Saglam, M.; Arslan, U.; Kayis, S. A.; Kebapcilar, L.; Loos, B. G.; Hakki, S. S.<br>Lasers in Medical Science 2020	No control group<br>No non-diabetic participants
Effect of diode laser application as an adjunct to nonsurgical periodontal therapy on the reduction of red complex microorganisms in type 2 diabetics with chronic periodontitis<br>Kocak, E.; Saglam, M.; Arslan, U.; Kayis, S. A.; Kebapcilar, L.; Loos, B. G.; Hakki, S. S.<br>Lasers in Medical Science 2020	Duplicate study<br>Study has been included in full text screening twice
Clinical, Microbial, and Immune Responses Observed in Patients With Diabetes After Treatment for Gingivitis: A Three-Month Randomized Clinical Trial<br>Raslan, S. A.; Cortelli, J. R.; Costa, F. O.; Aquino, D. R.; Franco, G. C.; Cota, L. O.; Gargioni-Filho, A.; Cortelli, S. C.<br>Journal of Periodontology 2015	Wrong population<br>Gingivitis rather than periodontitis

Is a single session of antimicrobial photodynamic therapy as an adjuvant to non-surgical scaling and root planing effective in reducing periodontal inflammation and subgingival presence of<i> Porphyromonas gingivalis </i>and <i>Aggregatibacter actinomycetemcomitans</i> in patients with periodontitis?<br>Aabed, K.; Moubayed, N.; Binshabaib, M. S.; Alharthi, S. S.<br>Photodiagnosis and Photodynamic Therapy 2022	Wrong population<br>No diabetic participants
Isolation and antibiotic susceptibility of anaerobic bacteria of periodontitis in diabetic and non-diabetic patients in Basrah Province, South of Iraq<br>Al-Farhan, S. R.; Al-Mussawi, A. A.; Al-Abdulla, A. A.<br>Indian Journal of Forensic Medicine and Toxicology 2019	Wrong Intervention<br>No intervention, only samples collected
Indocyanine-mediated antimicrobial photodynamic therapy promotes superior clinical effects in stage III and grade C chronic periodontitis among controlled and uncontrolled diabetes mellitus: A randomized controlled clinical trial<br>Al-Momani<br>Photodiagnosis and Photodynamic Therapy 2021	No control group<br>No non-diabetic participants
Evaluation of adjunctive systemic doxycycline with non-surgical periodontal therapy within type 2 diabetic patients<br>Al-Nowaiser, A. M.; Al-Zoman, H.; Baskaradoss, J. K.; Robert, A. A.; Al-Zoman, K. H.; Al-Sohail, A. M.; Al-Suwyed, A. S.; Ciancio, S. G.; Al-Mubarak, S. A.<br>Saudi Medical Journal 2014	No control group<br>No non-diabetic participants
Next generation sequencing of oral microbiota in Type 2 diabetes mellitus prior to and after neem stick usage and correlation with serum monocyte chemoattractant-1<br>Anbalagan, R.; Srikanth, P.; Mani, M.; Barani, R.; Seshadri, K. G.; Janarthanan, R.<br>Diabetes Research and Clinical Practice 2017	Wrong intervention<br>No treatment for non-diabetic participants
The effect of antimicrobial photodynamic therapy on periodontal disease and glycemic control in patients with type 2 diabetes mellitus<br>Brinar, S.; Skvarča, A.; Gašpirc, B.; Schara, R.<br>Clinical Oral Investigations 2023	No control group<br>No non-diabetic participants
Diode Laser - A Novel Therapeutic Approach in the Treatment of Chronic Periodontitis in Type 2 Diabetes Mellitus Patients: A Prospective Randomized Controlled Clinical Trial<br>Chandra, S.; Shashikumar, P.<br>Journal of Lasers in Medical Sciences 2019	No control group<br>No non-diabetic participants
Effects of multiple sessions of antimicrobial photodynamic therapy (aPDT) in the treatment of periodontitis in patients with uncompensated type 2 diabetes: A randomized controlled clinical study	No control group<br>No non-diabetic participants

Cláudio, M. M.; Nuernberg, M. A. A.; Rodrigues, J. V. S.; Belizário, L. C. G.; Batista, J. A.; Duque, C.; Garcia, V. G.; Theodoro, L. H.<br>Photodiagnosis and Photodynamic Therapy 2021	
Changes in Oral Subgingival Microbial Distribution and Community Structure in CP-T2DM Patients Before and After Combined Periodontal-Endodontic Treatment<br>Ding, Y.; Liu, Y.<br>Alternative Therapies in Health and Medicine 2023	Wrong intervention<br>Combined endodontic and periodontic therapy instead of non-surgical periodontal therapy
Avaliação clínica e microbiológica do uso sistêmico de doxiciclina como coadjuvante à terapia periodontal básica no tratamento de indivíduos com diabetes tipo 2 e doença periodontal crônica<br>do Monte Ribeiro Busato, Priscilla; Toyama, Gisele; Colet, Fernanda; Karin Vendrame, Taline; Andressa Felipetti, Francielly; Peruzzo Lopes, Claudia Camila; Gioppo, Nereida; Augusto Nassar, Carlos<br>Journal of Clinical Dentistry and Research 2017	Unable to access full text<br>Unable to access the full text article despite contacting authors
Clinical and microbiological effects of scaling and root planing, metronidazole and amoxicillin in the treatment of diabetic and non-diabetic subjects with periodontitis: A cohort study<br>Duarte, P. M.; Feres, M.; Yassine, L. L. S.; Soares, G. M. S.; Miranda, T. S.; Faveri, M.; Retamal-Valdes, B.; Figueiredo, L. C.<br>Journal of Clinical Periodontology 2018	Duplicate study<br>Study has been included in full text screening twice
A comparative clinical, microbiological and glycemic analysis of photodynamic therapy and Lactobacillus reuteri in the treatment of chronic periodontitis in type-2 diabetes mellitus patients<br>Elsadek, M. F.; Ahmed, B. M.; Alkhawtani, D. M.; Zia Siddiqui, A.<br>Photodiagnosis and Photodynamic Therapy 2020	No control group<br>No non-diabetic participants
Full-mouth scaling and root planing in type 2 diabetic subjects: one-year microbiological outcomes<br>Figueiredo, L. C.; Souza, D. C.; Santos, V. R.; Miranda, T. S.; Feres, M.; Faveri, M.; Duarte, P. M.<br>Australian Dental Journal 2014	No control group<br>No non-diabetic participants
The effect of non-surgical periodontal therapy on glycemic control and bacterial levels in a Mexican-American population with type 2 diabetes<br>Gay, I. C.; Tribble, G.; Katancik, J.<br>Journal of Clinical and Translational Science 2014	No control group<br>No non-diabetic participants
The interplay between gingival crevicular fluid microbiome and metabolomic profile in intensively treated people with type 1 diabetes - a combined metagenomic/metabolomic approach cross-sectional study<br>Gregorczyk-Maga, I.; Kania, M.; Dąbrowska, M.; Samborowska, E.; Żeber-Lubecka, N.; Kulecka, M.; Klupa, T.<br>Frontiers in Endocrinology 2024	Wrong population<br>No patients with periodontitis

The interplay between gingival crevicular fluid microbiome and metabolomic profile in intensively treated people with type 1 diabetes - a combined metagenomic/metabolomic approach cross-sectional study<br>Gregorczyk-Maga, I.; Kania, M.; Dąbrowska, M.; Samborowska, E.; Żeber-Lubecka, N.; Kulecka, M.; Klupa, T.<br>Frontiers in Endocrinology 2024	Duplicate study<br>Study has been included in full text screening twice
Study on the dynamic changes of oral microbiota in type 2 diabetes patients with periodontitis after initial periodontal therapy<br>Song, W.; Kang, W.; Liu, X.; Sun, L.; Feng, Q.; Ge, S.<br>Chinese Journal of Stomatology 2022	Unable to access full text<br>Unable to access the full text article despite contacting authors
Study on the dynamic changes of oral microbiota in type 2 diabetes patients with periodontitis after initial periodontal therapy<br>Song, W.; Kang, W.; Liu, X.; Sun, L.; Feng, Q.; Ge, S.<br>Chinese Journal of Stomatology 2022	Duplicate study<br>Study has been included in full text screening twice
CLINICAL AND MICROBIOLOGICAL ASSESSMENT OF NON-SURGICAL TREATMENT OF CHRONIC PERIODONTITIS IN CONTROLLED AND UNCONTROLLED TYPE 2 DIABETIC PATIENTS<br>Mirnić, J.; Đurić, M.; Nikolić, N.; Veljović, T.; Gušić, I.; Petrović, Đ; Milašin, J.<br>Acta Clinica Croatia 2022	Duplicate study<br>Study has been included in full text screening twice
Novel 20% doxycycline-loaded PLGA nanospheres as adjunctive therapy in chronic periodontitis in type-2 diabetics: randomized clinical, immune and microbiological trial<br>Lecio, G.; Ribeiro, F. V.; Pimentel, S. P.; Reis, A. A.; da Silva, R. V. C.; Nociti-Jr, F.; Moura, L.; Duek, E.; Casati, M.; Casarin, R. C. V.<br>Clinical Oral Investigations 2020	Duplicate study<br>Study has been included in full text screening twice
Novel 20% doxycycline-loaded PLGA nanospheres as adjunctive therapy in chronic periodontitis in type-2 diabetics: randomized clinical, immune and microbiological trial<br>Lecio, G.; Ribeiro, F. V.; Pimentel, S. P.; Reis, A. A.; da Silva, R. V. C.; Nociti-Jr, F.; Moura, L.; Duek, E.; Casati, M.; Casarin, R. C. V.<br>Clinical Oral Investigations 2020	No control group<br>No non-diabetic participants
Effect of adjuvant probiotic therapy (Lactobacillus reuteri) in the treatment of periodontitis associated with diabetes mellitus: clinical, controlled, and randomized study<br>Jardini, Maria Aparecida Neves; Pedroso, Juliana Fatima; Ferreira, Camila Lopes; Nunes, Camilla Magnoni Moretto; Reichert, Cadiele Oliana; Aldin, Marlene Nunez; Figueiredo Neto, Antonio Martins; Levy, Debora; Damasceno, Nagila Raquel Teixeira	Duplicate study<br>Study has been included in full text screening twice

Clinical Oral Investigations 2024	
Effect of adjuvant probiotic therapy (Lactobacillus reuteri) in the treatment of periodontitis associated with diabetes mellitus: clinical, controlled, and randomized study<br>Jardini, Maria Aparecida Neves; Pedroso, Juliana Fatima; Ferreira, Camila Lopes; Nunes, Camilla Magnoni Moretto; Reichert, Cadiele Oliana; Aldin, Marlene Nunez; Figueiredo Neto, Antonio Martins; Levy, Debora; Damasceno, Nagila Raquel Teixeira<br>Clinical Oral Investigations 2024	No control group<br>No non-diabetic participants
MICROBIOLOGICAL EFFECTS OF PERIODONTAL THERAPY<br>PLUS AZITHROMYCIN IN PATIENTS WITH DIABETES:<br>RESULTS FROM A RANDOMIZED CLINICAL TRIAL<br>Hincapié, J. P.; Castrillón, C. A.; Yepes, F. L.; Roldan, N.; Becerra, M. A.; Moreno, S. M.; Consuegra, J.; Contreras, A.; Botero, J. E.<br>Acta odontológica Latinoamericana 2014	No control group<br>No non-diabetic participants
Relationship of Porphyromonas gingivalis with glycemic level in patients with type 2 diabetes following periodontal treatment<br>Makiura, N.; Ojima, M.; Kou, Y.; Furuta, N.; Okahashi, N.; Shizukuishi, S.; Amano, A.<br>Oral Microbiology and Immunology 2008	No control group<br>No non-diabetic participants
Effects of mouthwash on periodontal pathogens and glycemic control in patients with type 2 diabetes mellitus<br>Matayoshi, S.; Tojo, F.; Suehiro, Y.; Okuda, M.; Takagi, M.; Ochiai, M.; Kadono, M.; Mikasa, Y.; Okawa, R.; Nomura, R.; Itoh, Y.; Itoh, N.; Nakano, K.<br>Scientific Reports 2024	No control group<br>No non-diabetic participants
Potential short-term shift in oral microbiota of patients with stage III-IV periodontitis and type 2 diabetes treated by non-surgical periodontal therapy<br>Xu, X.; He, L.; Huo, P.; Jin, D.; Zhu, Y.; Lu, H.; Meng, H.<br>Journal of Dental Sciences 2025	No control group<br>No non-diabetic participants
Efficacy of scaling and root planing with and without adjunct Nd:YAG laser therapy on glucose control and periodontal microecological imbalance in periodontitis patients with type 2 diabetes mellitus: a randomized controlled trial<br>Zhang, Yifei; Tang, Pengzhou; Yang, Qi; Li, Chen; Li, Lu; Han, Minxuan<br>Clinical Oral Investigations 2024	No control group<br>No non-diabetic participants
Changes of saliva microbiota in the onset and after the treatment of diabetes in patients with periodontitis<br>Yang, Y.; Liu, S.; Wang, Y.; Wang, Z.; Ding, W.; Sun, X.; He, K.; Feng, Q.; Zhang, X.	Wrong Intervention

Aging 2020	Treatment for diabetes rather than periodontitis
Benefits of non-surgical periodontal treatment in patients with type 2 diabetes mellitus and chronic periodontitis: A randomized controlled trial<br>Mauri-Obradors, E.; Merlos, A.; Estrugo-Devesa, A.; Jané-Salas, E.; López-López, J.; Viñas, M<br>Journal of Clinical Periodontology 2018	No control group<br>No non-diabetic participants
Metronidazole and amoxicillin as adjuncts to scaling and root planing for the treatment of type 2 diabetic subjects with periodontitis: 1-year outcomes of a randomized placebo-controlled clinical trial<br>Miranda, T. S.; Feres, M.; Perez-Chaparro, P. J.; Faveri, M.; Figueiredo, L. C.; Tamashiro, N. S.; Bastos, M. F.; Duarte, P. M.<br>Journal of Clinical Periodontology 2014	No control group<br>No non-diabetic participants
Periodontal conditions and prevalence of putative periodontopathogens and Candida spp. in insulin-dependent type 2 diabetic and non-diabetic patients with chronic periodontitis--a pilot study<br>Sardi, J. C.; Duque, C.; Camargo, G. A.; Hofling, J. F.; Gonçalves, R. B.<br>Archives of Oral Biology 2011	Wrong Intervention<br>No non-surgical periodontal therapy, this study only evaluated the presence of putative periodontopathogenic bacteria in periodontal pockets of patients with and without T2DM and chronic periodontitis
Amoxicillin Plus Metronidazole Therapy for Patients with Periodontitis and Type 2 Diabetes: A 2-year Randomized Controlled Trial<br>Tamashiro, N. S.; Duarte, P. M.; Miranda, T. S.; Maciel, S. S.; Figueiredo, L. C.; Faveri, M.; Feres, M.<br>Journal of Dental Research 2016	No control group<br>No non-diabetic participants
Effect of oral multispecies probiotic on wound healing, periodontitis and quality of life on patients with diabetes<br>Stuermer, E. K.; Bang, C.; Giessler, A.; Smeets, R.; Janke, T. M.; Seki, F. D.; Debus, E. S.; Franke, A.; Augustin, M.<br>Journal of Wound Care 2024	No control group<br>No non-diabetic participants
Effects of doxycycline on clinical, microbiological and immunological parameters in well-controlled diabetes type-2 patients with periodontal disease: a randomized, controlled clinical trial<br>Tsalikis, Lazaros; Sakellari, Dimitra; Dagalis, Panagiotis; Boura, Panagiota; Konstantinidis, Antonios<br>Journal of Clinical Periodontology 2014	No control group<br>No non-diabetic participants

Supplementary Table S5. Diabetes characteristics and glycaemic control of participants in the included studies

Author, year	Diabetes type; n with diabetes / n without diabetes	Criterion used to diagnose diabetes	Baseline HbA1c, diabetic group (%)	Baseline HbA1c, non-diabetic group (%)	Glycaemic control categories and thresholds	Diabetes duration; glucose-lowering therapy	HbA1c reported after NSPT
Almeida et al., 2019	T2DM; 26 / 28	Diagnosis of T2DM ≥2years established by a physician, DM treatment with insulin/oral hypoglycemiant, HbA1c ≥6.5%, fasting plasma glucose ≥126mg/dL	7.5 ± 0.9	5.9 ± 0.6	Not stratified	NR	NR
Buzinin et al., 2014	T1DM; 20 / 21	NR - mentioned that subjects of the test group should have been diagnosed with type 1 diabetes mellitus	9.24 ± 2.34	NR	Not stratified	(mean ± SD)<br><7 years: 3.12 ± 2.01<br>7-12 years: 8.80 ± 0.83<br><12 years: 29.0 ± 8.83	Only for DM group: 8.93 ± 2.35
Christgau et al., 1998	T1DM and T2DM; 20 / 20	NR:<br>Diabetics were selected from the pool of patients referred to the Endocrine Outpatients' Department. Department of Internal Medicine I of the University of Regensburg	Median value: 6.5<br>Range: 4.4-10.6	Median value: 4.3	Not stratified; T1DM and T2DM analysed together	Median duration of disease: 11.5 years	DM: 6.7%<br><br>ND: 4.7

Author, year	Diabetes type; n with diabetes / n without diabetes	Criterion used to diagnose diabetes	Baseline HbA1c, diabetic group (%)	Baseline HbA1c, non-diabetic group (%)	Glycaemic control categories and thresholds	Diabetes duration; glucose-lowering therapy	HbA1c reported after NSPT
da Cruz et al., 2008	T2DM; 10 / 10	Insulin supplementation confirmed by an endocrinologist	9.23 ± 2.60	5.88 ± 0.16	Not stratified	Mean time since initial diagnosis of diabetes: 18.10 ± 11.37	ND: 5.82 ± 2.60 DM: 9.4 ± 2.53
Devina et al., 2024	T2DM; 18 / 18	Those with HbA1c levels below 6.5% will be categorized into the non-DM group, whereas those with results above 6.5% will be classified into the DM group	DM (placebo): 7.378 ± 0.7949<br><br>DM (HA gel): 7.278 ± 0.6119	ND (placebo): 5.356 ± 0.6085<br><br>ND (HA gel): 5.467 ± 0.5831	Not stratified	NR	NR
Duarte et al., 2018	T2DM; 29 / 29	Diagnosis of type 2 DM for ≥5 years, DM treatment with diet and insulin supplementation or oral hypoglycaemic agents and, glycated haemoglobin (HbA1c) levels ≥6.5% and ≤11%	NR	NR	Not stratified	Diagnosis of type 2 DM for ≥5 years	NR

Author, year	Diabetes type; n with diabetes / n without diabetes	Criterion used to diagnose diabetes	Baseline HbA1c, diabetic group (%)	Baseline HbA1c, non-diabetic group (%)	Glycaemic control categories and thresholds	Diabetes duration; glucose-lowering therapy	HbA1c reported after NSPT
Hropot Plesko et al., 2021	T1DM (n = 11) and T2DM (n = 11); 22 / 11	NR	T2DM: 7.99 ± 1.02<br><br>T1DM: 7.01 ± 0.77	ND: 5.38 ± 0.26	Stratified by diabetes type, not by glycaemic control	NR	T2DM: 7.64 ± 0.79<br><br>T1DM: 6.95 ± 0.82<br><br>ND: 5.29 ± 0.21
Lakhani et al., 2019	T2DM; 90 / 30	NR	NR	NR	Three strata of 30: good control (HbA1c < 5%), moderate control (HbA1c < 7%) and poor control (HbA1c > 8%), as reported	NR	NR
Mirnić et al., 2021	T2DM; 61 / 31	NR	Well controlled DM (HbA1c<7%) 6.15±0.45<br><br>Poorly controlled DM (HbA1c≥7%) 8.35±1.42	ND: 5.51±0.32	Well-controlled HbA1c <7% (n = 29) and poorly controlled HbA1c ≥7% (n = 32); threshold	NR	Well controlled DM (HbA1c<7%) 6.54±1.15<br><br>Poorly controlled DM (HbA1c≥7%) 7.9±1.45<br><br>ND: 5.46±0.27

Author, year	Diabetes type; n with diabetes / n without diabetes	Criterion used to diagnose diabetes	Baseline HbA1c, diabetic group (%)	Baseline HbA1c, non-diabetic group (%)	Glycaemic control categories and thresholds	Diabetes duration; glucose-lowering therapy	HbA1c reported after NSPT
Rajpurohit et al., 2024	T2DM; 15 / 15 (plus 15 periodontally healthy)	HbA1c >7%	NR	NR	Not stratified	Diabetes diagnosis ≥5 years	NR
Rode et al., 2021	T2DM; 20 / 20 (plus 20 periodontally healthy)	HbA1c between 6.5%-7%	HbA1c between 6.5%-7%	HbA1c<6.5%	Not stratified	Controlled T2DM for 5-10 years	NR
Sambashivaiah et al., 2011	T2DM; 12 / 12 (plus 12 periodontally healthy)	Known cases of type II DM in group III were assessed by random blood sugar level (RBS) and questionnaire	NR	NR	Not stratified	NR	NR
Silva-Boghossian et al., 2014	T2DM; 20 / 20	HbA1c ≥7%	9.43 ± 1.80	NR	Single diabetic group with inadequate metabolic control; HbA1c ≥7%	Mean DM duration of 9.85 ± 7.10 years	DM: 9.03 ± 1.94
Wang et al., 2023	T2DM; 11 / 11	NR	7.3 ± 1.3	5.5 ± 0.3	Not stratified	Diagnosed with T2DM for at least the past 1 year	DM: 6.7 ± 0.8<br><br>ND: 5.6 ± 0.4

HbA1c values are given as reported in the original publications (mean ± SD unless otherwise indicated). NR, not reported; ND, non-diabetic; NSPT, non-surgical periodontal therapy; T1DM, type 1 diabetes mellitus; T2DM, type 2 diabetes mellitus. Participant numbers refer to participants with periodontitis and exclude periodontally healthy control groups.

Table S6. Periodontal diagnostic criteria, disease severity and treatment protocol of the included studies

Author, year	Classification system	Case definition / inclusion threshold	Reported severity and extent	Baseline mean PPD (mm), DM / ND	Baseline mean CAL (mm), DM / ND	NSPT delivery and adjunct	Protocol category
Almeida et al., 2019	Mild periodontitis: ≥ 2 interproximal sites with attachment loss (AL) ≥ 3 mm and ≥ 2 interproximal sites with PD ≥ 4mm, in different tooth, or one site with ≥ 5 mm. Moderate periodontitis: ≥2 interproximal sites with AL ≥ 4 mm and ≥ 2 interproximal sites with PD ≥ 5 mm in different tooth	General inclusion criteria were ≥ 35 years of age, at least 15 teeth (excluding third molars and teeth with advanced decay indicated for extraction) and mild-to-moderate periodontitis	Mild to moderate chronic periodontitis	DM: 2.84 ± 0.67<br><br>ND: 2.79 ± 0.74	DM: 3.83 ± 2.02<br><br>ND: 2.36 ± 1.57	Full-mouth disinfection: FMSRP with 0.12% CHX rinse, 1% CHX gel pocket irrigation and tongue brushing; home rinsing with 0.12% CHX	Mechanical + adjunct (CHX)

Author, year	Classification system	Case definition / inclusion threshold	Reported severity and extent	Baseline mean PPD (mm), DM / ND	Baseline mean CAL (mm), DM / ND	NSPT delivery and adjunct	Protocol category
Buzinin et al., 2014	All subjects: minimum 12 natural teeth and diagnosed with periodontal disease: ≥5 sites of pockets of ≥5 mm and probing attachment level (PAL) ≥3 mm	All subjects had to have a minimum of 12 natural teeth and diagnosed with periodontal disease with ≥5 sites of pockets of ≥5 mm and probing attachment level (PAL) ≥3 mm	Moderate to advanced chronic periodontitis	Structured differently: Number of sites<br>DM group:<br>PPD<4mm<br>79.69 ± 15.31<br>PPD 4-6mm<br>19.68 ± 13.80<br>PPD>6mm<br>0.68 ± 1.58<br><br>ND group:<br>PPD<4mm<br>74.96 ± 11.49<br><br>PPD 4-6mm<br>27.35 ± 13.28<br><br>PPD>6mm<br>1.78 ± 2.80	Structured differently: Number of sites (probing attachment level – PAL)<br>DM group:<br>PAL<4mm<br>7.53 ± 9.98<br><br>PAL4-6mm<br>26.90 ± 18.85<br><br>PAL>6mm<br>1.85 ± 3.26<br><br>ND group:<br>PAL<4mm<br>9.24 ± 5.5<br><br>PAL4-6mm<br>34.38 ± 10.69<br><br>PAL>6mm<br>3.12 ± 5.77	Single-session full-mouth debridement (ultrasonic scaler and Gracey curettes)	Mechanical only

**Author, year**	**Classification system**	**Case definition / inclusion threshold**	**Reported severity and extent**	**Baseline mean PPD (mm), DM / ND**	**Baseline mean CAL (mm), DM / ND**	**NSPT delivery and adjunct**	**Protocol category**
Christgau et al., 1998	NR	Min. age of 30 years, ≥16 remaining teeth, moderate - advanced periodontitis with signs of an active inflammation (bleeding or pus on probing), ≥6 teeth with PPD ≥4 mm, no evidence of systemic diseases other than diabetes being a risk factor for periodontitis, no antibiotic therapy within the preceding 6 months, no need for endocarditis prophylaxis	Moderate to advanced chronic periodontitis	Structured differently (as a %):<br>DM:<br>PPD 1-3mm (%): 59.9<br>PPD 4-5mm (%): 29.9<br>PPD ≥6mm (%): 12.0<br><br>ND:<br>PPD 1-3mm (%): 54.2<br>PPD 4-5mm (%): 27.6<br>PPD ≥6mm (%): 14.0	Structured differently (as a %): (probing attachment level – PAL)<br>DM:<br>PAL 1-3mm (%): 39.7<br>PAL 4-5mm (%): 33.4<br>PAL ≥6mm (%): 16.6<br><br>ND:<br>PAL 1-3mm (%): 41.5<br>PAL 4-5mm (%): 35.3<br>PAL ≥6mm (%): 20.7	Initial therapy (OHI, removal of overhangs, extraction of hopeless teeth, splinting), then quadrant-wise SRP with hand instruments under LA; pockets irrigated with 0.2% CHX solution and 1% CHX gel	Mechanical + adjunct (CHX)

Author, year	Classification system	Case definition / inclusion threshold	Reported severity and extent	Baseline mean PPD (mm), DM / ND	Baseline mean CAL (mm), DM / ND	NSPT delivery and adjunct	Protocol category
da Cruz et al., 2008	Clinical diagnosis of generalized chronic periodontitis defined by probing depth (PD) ≥5 mm in ≥10 teeth, radiographic bone loss ranging from 30% to 50%, and ≥20 teeth	Exclusion criteria were the use of antibiotics during the previous 6 months, pregnancy, smoking, systemic diseases, or the use of immune-suppressive medication, phenytoin, cyclosporin, calcium channel blockers, or any medication that could interfere with the periodontium or the response to periodontal therapy	Generalised chronic periodontitis	DM: 5.72 ± 1.13<br><br>ND: 4.79 ± 0.9	DM: 4.49 ± 0.7<br><br>ND: 4.03 ± 0.37	Single-session FMSRP under LA (approximately 2 h) with OHI	Mechanical only

Author, year	Classification system	Case definition / inclusion threshold	Reported severity and extent	Baseline mean PPD (mm), DM / ND	Baseline mean CAL (mm), DM / ND	NSPT delivery and adjunct	Protocol category
Devina et al., 2024	2018 World Workshop classification	Participants must be diagnosed with periodontitis according to European Federation of Periodontology (EFP) 2017[23] Interdental clinical attachment loss (CAL) is detectable at ≥ 2 nonadjacent teeth, or buccal CAL of ≥3mm with pocketing of more than 3mm is detectable at ≥ 2 teeth	NR	DM (placebo): 4.89 ± 0.928<br><br>DM (HA gel): 4.78 ± 0.972<br>ND (placebo): 4.56 ± 0.726<br><br>ND (HA gel): 5.11 ± 0.782	DM (placebo): 6.11 ± 1.691<br><br>DM (HA gel): 5.33 ± 2.398<br>ND (placebo): 5.11 ± 1.833<br><br>ND (HA gel): 5.0 ± 0.866	FMSRP with OHI; randomised to 0.2% hyaluronic acid gel or placebo gel	Mechanical + adjunct (HA); placebo arm = mechanical only

Author, year	Classification system	Case definition / inclusion threshold	Reported severity and extent	Baseline mean PPD (mm), DM / ND	Baseline mean CAL (mm), DM / ND	NSPT delivery and adjunct	Protocol category
Duarte et al., 2018	Armitage (1999)	≥30 years of age, at least 15 teeth (excluding third molars and teeth with advanced decay indicated for extraction); more than 30% of the sites with PD and clinical attachment level (CAL) ≥4 mm and bleeding on probing (BoP) at baseline, and a minimum of six teeth with at least one site with PD and CAL ≥5 mm	Severe chronic periodontitis	DM: 3.64 ± 0.59<br><br>ND: 3.69 ± 0.63	DM: 4.59 ± 1.23<br><br>ND: 4.13 ± 0.83	SRP with systemic metronidazole 400 mg and amoxicillin 500 mg three times daily for 14 days	Mechanical + adjunct (systemic antibiotics)
Hropot Plesko et al., 2021	2018 World Workshop classification	All patients were diagnosed with at least stage II periodontitis, grades B or C (chronic periodontitis).	At least stage II, grade B or C	T2DM: 3.05 ± 1.23<br><br>T1DM: 3.02 ± 1.08<br><br>ND: 3.18 ± 1.39	T2DM: 3.40 ± 1.45<br><br>T1DM: 3.11 ± 1.17<br><br>ND: 3.42 ± 1.55	Full-mouth disinfection: supragingival plaque removal, SRP, tongue scraping and pocket rinsing with 0.12% CHX; home rinsing with 0.12% CHX for 2 weeks	Mechanical + adjunct (CHX)

Author, year	Classification system	Case definition / inclusion threshold	Reported severity and extent	Baseline mean PPD (mm), DM / ND	Baseline mean CAL (mm), DM / ND	NSPT delivery and adjunct	Protocol category
Lakhani et al., 2019	NR	Patients with chronic periodontitis (mild to moderate with up to 4 mm (CAL) clinical attachment loss and with the presence of true periodontal pocket (up to 6 mm) in ≥30% of sites in the mouth	Mild to moderate chronic periodontitis	NR	NR	Ultrasonic SRP	Mechanical only
Mirnić et al., 2021	Periodontitis was defined as minimum ≥2 sites with clinical attachment level (CAL) ≥3 mm and ≥2 sites with probing depth (PD) ≥4 mm at different teeth or 1 site with PD ≥5 mm2	Age 30-70 years, type 2 DM treated with oral antidiabetic agents, and clinically diagnosed chronic periodontitis	Chronic periodontitis; NO SEVERITY LISTED	Well controlled DM (HbA1c <7%) 2.09±0.51<br><br>Poorly controlled DM (HbA1c ≥7%) 2.11±0.47<br><br>ND: 2.38±0.60	Well controlled DM (HbA1c <7%) 2.81±0.98<br><br>Poorly controlled DM (HbA1c ≥7%) 2.62±1.36<br><br>ND: 2.32±1.39	SRP (ultrasonic scaler and Gracey curettes) with OHI	Mechanical only
Rajpurohit et al., 2024	NR	NR	Chronic periodontitis; NO SEVERITY LISTED	NR	NR	SRP	Mechanical only

**Author, year**	**Classification system**	**Case definition / inclusion threshold**	**Reported severity and extent**	**Baseline mean PPD (mm), DM / ND**	**Baseline mean CAL (mm), DM / ND**	**NSPT delivery and adjunct**	**Protocol category**
Rode et al., 2021	≥20 natural teeth with moderate-to-severe clinical attachment loss with >30% of sites involved	Patients aged more than 35 years with the presence of at least twenty natural teeth with moderate-to-severe clinical attachment loss with >30% of sites involved	Moderate to severe chronic periodontitis	Periodontally healthy: 1.26 ± 0.22<br><br>Chronic periodontitis (CP): 4.62 ± 0.78<br><br>CP + T2DM: 6.28 ± 1.52	Periodontally healthy: 0<br><br>Chronic periodontitis (CP): 5.28 ± 0.80<br><br>CP + T2DM: 7.14 ± 1.59	NSPT; SRP	Mechanical only
Sambashivaiah et al., 2011	Periodontal Disease Classification System of the American Academy of Periodontology (1999)	PPD > 5 mm<br><br>Chronic periodontitis patients with minimum of 20 teeth and average probing depth of >5 mm with and without history of Type II diabetes mellitus were included in the study	Chronic periodontitis	Healthy 1.33±0.33<br><br>Chronic periodontitis 7.91±0.91<br><br>Chronic periodontitis + T2DM 8.08±1.31	NR	FMSRP under LA with home-care instruction	Mechanical only

Author, year	Classification system	Case definition / inclusion threshold	Reported severity and extent	Baseline mean PPD (mm), DM / ND	Baseline mean CAL (mm), DM / ND	NSPT delivery and adjunct	Protocol category
Silva-Boghossian et al., 2014	At least 4 teeth with one or more sites with probing depth (PD) ≥ 5 mm, clinical attachment level (CAL) ≥ 4 mm, and bleeding on probing (BOP)	All participants had a diagnosis of chronic periodontitis, at least 15 teeth (except third molars), and moderate to severe CP—i.e., at least 4 teeth with one or more sites with probing depth (PD) ≥ 5 mm, clinical attachment level (CAL) ≥ 4 mm, and bleeding on probing (BOP)	Moderate to severe chronic periodontitis	DM: 4.2 ± 0.9<br><br>ND: 3.6 ± 0.6	DM: 4.9 ± 1.5<br><br>ND: 4.3 ± 1.0	OHI and SRP in four weekly 1-h sessions under LA; professional plaque control twice monthly for 3 months	Mechanical only
Wang et al., 2023	NR	NR	Chronic periodontitis; NO SEVERITY LISTED	DM: 3.2 ± 0.8<br><br>ND: 3.3 ± 0.9	DM: 3.5 ± 0.6<br><br>ND: 2.9 ± 1.4	SRP with OHI (modified Bass technique); OHI repeated at 3 months	Mechanical only

The classification system and case definition are as reported by the original authors. Baseline PPD and CAL are group means as reported; values at each follow-up are given in Supplementary Tables S4 and S5. Protocol category: ‘mechanical only’ denotes subgingival debridement (SRP or full-mouth debridement) with oral hygiene instruction and without an antimicrobial or other adjunctive agent. CAL, clinical attachment level; CHX, chlorhexidine; DM, diabetic; FMSRP, full-mouth scaling and root planing; HA, hyaluronic acid; LA, local anaesthesia; ND, non-diabetic; OHI, oral hygiene instruction; PPD, probing pocket depth; SRP, scaling and root planing.

Table S7. Summary of changes in periodontal probing depth at baseline and following non-surgical periodontal therapy

Author, Year	PPD measure	Baseline	Post treatment change	Overall Summary
**Assessed prevalence of periodontal probing depth (PPD) %**				
Almeida et. al, 2019	% sites with PD ≥ 4 mm	Non-diabetic + PD: 20.8 ± 15.8<br>Diabetic + PD: 22.4 ± 19.1	3 months<br>Non-diabetic + PD:13.5 ± 12.2<br>Diabetic + PD: 12.9 ± 16.8<br>6 months<br>Non-diabetic + PD:11.3 ± 12.5<br>Diabetic + PD:16.6 ± 23.8<br>12 months<br>Non-diabetic + PD:9.30 ± 9.15<br>Diabetic + PD:9.97 ± 12.5	Following NSPT, there was a decrease in PD in both groups, with a slightly greater decrease reported in the Diabetic group at 3 months and 12 months following baseline
Buzinin et. al, 2014	% sites with PPD < 4	Non-diabetic with PD: 74.96 ± 11.49<br>Diabetic with PD: 79.69 ± 15.31	2 months<br>Non-diabetic with PD:92.69 ± 5.6<br>Diabetic with PD:95.73 ± 3.37<br>3 months<br>Non-diabetic with PD: 95.7 ± 4.13<br>Diabetic with PD: 97.76 ± 1.87	There was a decrease in PPD in both groups, with a slightly more pronounced decrease reported in the Diabetic group at all intervals.
	% sites with PPD 4-6mm	Non-diabetic: 27.35 ± 13.28<br>Diabetic: 19.68 ± 13.80	2 months<br>Non-diabetic: 6.44 ± 4.87<br>Diabetic: 4.07 ± 2.74<br>3 months<br>Non-diabetic: 4.10 ± 3.62<br>Diabetic: 2.26 ± 1.82	
	% sites with PPD >6mm	Non-diabetic: 1.78 ± 2.80<br>Diabetic: 0.68 ± 1.58	2 months<br>Non-diabetic:0.32 ± 0.73<br>Diabetic: 0.19 ± 0.74<br>3 months<br>Non-diabetic: 0.20 ± 0.69<br>Diabetic: 0.00 ± 0.00	

Christgau et. al, 1998	PPD 1-3mm (%) as a median %	Non-diabetic: 54.2<br>Diabetic: 59.9	<u>3 months</u><br>Non-diabetic:57.7<br>Diabetic: 62.9<br><u>4 months</u><br>Non-diabetic: 67.9<br>Diabetic: 70.8	Following NSPT, there was a decrease in PPD for both groups. The Diabetic group reported a greater decrease at all intervals.
	PPD 4-5mm (%) as a median %	Non-diabetic: 27.6<br>Diabetic: 29.9	<u>3 months</u><br>Non-diabetic: 28.5<br>Diabetic: 27.3<br><u>4 months</u><br>Non-diabetic: 27.5<br>Diabetic: 25.5	
	PPD ≥6mm (%) as a median %	Non-diabetic: 14.0<br>Diabetic: 12.0	<u>3 months</u><br>Non-diabetic:10.4<br>Diabetic: 6.9<br><u>4 months</u><br>Non-diabetic: 4.3<br>Diabetic: 2.8	
**Assessed mean probing depth**				
da Cruz et. al, 2008	Mean PPD (mm) ± SD	Non-diabetic: 4.79 ± 0.9<br>Diabetic: 5.72 ± 1.13	<u>3 months</u><br>Non-diabetic: 3.97 ± 0.82<br>Diabetic: 5.00 ± 1.29	Following NSPT, there was a decrease in PPD for both groups with both groups reporting a similar level of decrease 3 months post NSPT.
Devina et. al, 2024	Mean PPD (mm) ± SD	Non-diabetic + PD (placebo gel): 4.56 ± 0.726<br>Non-diabetic + PD (hyaluronic acid gel): 5.11 ± 0.782<br>Diabetic + PD (placebo gel): 4.89 ± 0.928<br>Diabetic + PD (hyaluronic acid gel): 4.78 ± 0.972	<u>4 weeks</u><br>Non-diabetic + PD (placebo gel):4.0 ± 1.0<br>Non-diabetic + PD (hyaluronic acid gel): 3.44 ± 0.8<br>Diabetic + PD (placebo gel): 4.0 ± 1.3<br>Diabetic + PD (hyaluronic acid gel): 3.78 ± 1.6	Following NSPT, there was a decrease in PPD for all groups with the Non-diabetic group using the hyaluronic acid gel reporting a slightly higher level of decrease at the 4 week interval.

Duarte et. al, 2018	Mean PPD (mm) ± SD	Non-diabetic: 3.69 ± 0.63<br>Diabetic: 3.64 ± 0.59	<u>3 months</u><br>Non-diabetic: 2.56 ± 0.38<br>Diabetic: 2.65 ± 0.18<br><u>6 months</u><br>Non-diabetic: 2.51 ± 0.37<br>Diabetic: 2.58 ± 0.22<br><u>12 months</u><br>Non-diabetic: 2.48 ± 0.39<br>Diabetic: 2.53 ± 0.22	Following NSPT, there was a similar level of decrease in PPD for both groups at all intervals.
	Mean number (± SD) of sites with PD ≥ 5 mm and PD ≥ 6 mm at baseline and follow-up visits	Non-diabetic: 33.55 ± 18.61<br>Diabetic: 32.24 ± 18.68	<u>3 months</u><br>Non-diabetic: 4.72 ± 6.21<br>Diabetic: 4.55 ± 3.30<br><u>6 months</u><br>Non-diabetic: 4.23 ± 5.17<br>Diabetic: 3.32 ± 2.63<br><u>12 months</u><br>Non-diabetic: 4.12 ± 4.80<br>Diabetic: 4.00 ± 3.39	
Hropot Plesko et. al, 2021	Mean PPD (mm) ± SD	Non-diabetic: 3.18 ± 1.39<br>T1DM: 3.02 ± 1.08<br>T2DM: 3.05 ± 1.23	<u>3 months</u><br>Non-diabetic: 2.75 ± 1.08<br>T1DM: 2.94 ± 0.96<br>T2DM: 2.79 ± 1.09	Following NSPT, there was a similar level of decrease in PPD for all groups at the 3-month interval.
	PPD (0-4mm) (%)	Non-diabetic: 1474, 84.62%<br>T1DM: 1643, 91.58%<br>T2DM: 1314, 88.13%	<u>3 months</u><br>Non-diabetic: 1614, 92.76%<br>T1DM: 1694, 94.43%<br>T2DM: 1360, 92.90%	
	PPD 5-6mm (%)	Non-diabetic: 203, 11.65%<br>T1DM: 139, 7.75%<br>T2DM: 152 ± 10.19%	<u>3 months</u><br>Non-diabetic:104, 5.98%<br>T1DM: 93, 5.18%<br>T2DM: 90, 6.15%	
	PPD >6mm	Non-diabetic: 65, 3.73%<br>T1DM: 12, 0.67%<br>T2DM: 25 ± 1.68%	<u>3 months</u><br>Non-diabetic: 16, 1.26%<br>T1DM: 7, 0.39%	

			T2DM: 14, 0.96%	
Mirnić et. al, 2021	Mean PPD (mm) ± SD	Non-diabetic: 2.38±0.60<br>Well controlled diabetes: 2.09±0.51<br>Poorly controlled diabetes: 2.11±0.47	3 months<br>Non-diabetic: 2.05±0.52<br>Well controlled diabetes: 1.98±0.53<br>Poorly controlled diabetes: 2.03±0.35	Following NSPT, there was a similar level of decrease in PPD for all groups at the 3 month interval.
Rode et. al, 2021	Mean PPD (mm) ± SD	Non-PD: 1.26 ± 0.22<br>Non-diabetic and PD: 4.62 ± 0.78<br>T2DM and PD: 6.28 ± 1.52	3 months<br>Non-PD: 0.43 ± 0.35<br>Non-diabetic and PD: 2.58 ± 0.60<br>T2DM and PD: 4.01 ± 1.38	Following NSPT, there was a decrease in PPD for both groups with the Non-diabetic group reporting a slightly higher proportion of decrease at 3 months post NSPT.
Silva-Boghossian et. al, 2014	Mean PPD (mm) ± SD	Non-diabetic: 3.6 ± 0.6<br>Diabetic: 4.2 ± 0.9	3 months<br>Non-diabetic: 2.4 ± 0.3<br>Diabetic: 2.7 ± 0.4	Following NSPT, there was a similar level of decrease in PPD for both groups at the 3-month interval.
	PPD ≥ 5 mm (%)	Non-diabetic: 27.9 ± 11.2<br>Diabetic: 39.1 ± 24.2	3 months<br>Non-diabetic:4.2 ± 4.2<br>Diabetic:7.7 ± 7.3	
Wang et. al, 2023	Mean PPD (mm) ± SD	Non-diabetic: 3.3 ± 0.9<br>Diabetic: 3.2 ± 0.8	3 months<br>Non-diabetic: 2.5 ± 0.7<br>Diabetic: 2.8 ± 0.7	Following NSPT, there was a decrease in PPD for both groups with a slightly greater decrease reported for the non-diabetic group at the 3-month interval.

% sites with PD refer to the percentage of periodontal sites per patient within the indicated depth categories. Median (%) values represent the median proportion of sites per patient in each PPD category (with 25th/75th percentiles reported in the original studies). Mean PPD (mm) ± SD indicates mean probing depth in mm with standard deviation.
PD, periodontitis; ND, non-diabetic; DM, diabetes mellitus; T1DM, type 1 diabetes mellitus; T2DM, type 2 diabetes mellitus; PPD, probing pocket depth.

Supplementary Table S8. Summary of changes in periodontal clinical attachment level at baseline and following non-surgical periodontal therapy

Author, year	CAL measure	Baseline	Post NSPT change	Overall Summary
**Assessed mean clinical attachment loss (CAL) or periodontal attachment loss (PAL)**				
Almeida et. al, 2019	Mean CAL (mm) ± SD	Non-diabetic + PD: 2.36 ± 1.57<br>Diabetic + PD: 3.83 ± 2.02	<u>3 months</u><br>Non-diabetic and PD: 3.00 ± 1.28<br>Diabetic and PD: 3.67 ± 2.04<br><u>6 months</u><br>Non-diabetic and PD: 2.22 ± 1.25<br>Diabetic and PD: 3.79 ± 2.01<br><u>12 months</u><br>Non-diabetic and PD: 2.11 ± 1.20<br>Diabetic and PD: 3.52 ± 1.91	There was a decrease in CAL for both groups. The Non-diabetic group reported a slightly higher level of decrease at the 6 month and 12 month intervals. At the 3 month interval however, an increase in CAL was identified for the Non-diabetic group which reduced again at later intervals.
Buzinin et. al, 2014	PAL <4mm	Non-diabetic and PD: 9.24 ± 5.5<br>Diabetic and PD: 7.53 ± 9.98	<u>2 months</u><br>Non-diabetic+ PD: 16.13 ± 8.18<br>Diabetic and PD: 10.83 ± 13.60<br><u>3 months</u><br>Non-diabetic and PD: 19.56 ± 9.94<br>Diabetic and PD: 12.34 ± 14.93	There was a similar level of decrease in CAL reported among both groups at all time intervals.
	PAL 4-6mm	Non-diabetic and PD: 34.38 ± 10.69<br>Diabetic and PD: 26.90 ± 18.85	<u>2 months</u><br>Non-diabetic + PD: 16.35 ±10.28<br>Diabetic + PD: 9.89 ± 11.36<br><u>3 months</u><br>Non-diabetic + PD :11.97 ± 10.02<br>Diabetic + PD: 7.46 ± 9.22	
	PAL >6mm	Non-diabetic and PD: 3.12 ± 5.77<br>Diabetic + PD: 1.85 ± 3.26	<u>2 months</u><br>Non-diabetic and PD:1.50 ± 4.15<br>Diabetic and PD: 0.78 ± 1.64<br><u>3 months</u><br>Non-diabetic and PD:1.06 ± 3.29<br>Diabetic and PD:0.54 ± 1.67	

da Cruz et. al, 2008	Mean CAL (mm) ± SD	Non-diabetic and PD: 4.03 ± 0.37<br>Diabetic + PD: 4.49 ± 0.7	<u>3 months</u><br>Non-diabetic and PD:2.90 ± 0.26<br>Diabetic and PD:3.28 ± 0.54	There was a similar level of decrease in CAL reported among both groups at the 3 month interval.
Devina et. al, 2024	Mean CAL (mm) ± SD	Non-diabetic and PD (Placebo gel): 5.11 ± 1.833<br>Non-diabetic and PD (hyaluronic acid gel): 5.0 ± 0.866<br>Diabetic and PD (Placebo gel): 6.11 ± 1.691<br>Diabetic and PD (hyaluronic acid gel): 5.33 ± 2.398	<u>4 weeks</u><br>Non-diabetic and PD (Placebo gel): 4.56 ± 2.186<br>Non-diabetic and PD (hyaluronic acid gel): 3.22 ± 0.972<br>Diabetic and PD (Placebo gel): 5.33 ± 2.291<br>Diabetic and PD (hyaluronic acid gel):4.33 ± 3.162	There was a similar level of decrease in CAL reported across all groups at the 4 week interval.
Duarte et. al, 2018	Mean CAL (mm) ± SD	Non-diabetic and PD: 4.13 ± 0.83<br>Diabetic + PD: 4.59 ± 1.23	<u>3 months</u><br>Non-diabetic and PD: 3.19 ± 0.74<br>Diabetic and PD: 3.84 ± 0.96-<br><u>6 months</u><br>Non-diabetic:3.18 ± 0.77<br>Diabetic:3.67 ± 0.91<br><u>12 months</u><br>Non-diabetic:3.12 ± 0.72<br>Diabetic:3.73 ± 0.95	There was a slightly higher level of decrease in CAL reported for the Non-diabetic group from baseline to 12 months. At the 12 month interval, the CAL for the Diabetic group increase slightly in comparison to the 6 month interval, however it was still a decrease when compared to baseline.
Hropot Plesko et. al, 2021	Mean CAL (mm) ± SD	Non-diabetic + PD: 3.42 ± 1.55<br>T1DM + PD: 3.11 ± 1.17<br>T2DM + PD: 3.40 ± 1.45	<u>3 months</u><br>Non-diabetic and PD: 3.00 ± 1.28<br>T1DM and PD: 3.02 ± 1.06<br>T2DM and PD:3.16 ± 1.38	There was a slightly higher level of decrease in CAL for the Non-diabetic group at the 3 month interval.
Mirnić et. al, 2021	Mean CAL (mm) ± SD	Non-diabetic and PD: 2.81±0.98<br>Well controlled diabetes and PD: 2.81±0.98<br>Poorly controlled diabetes and PD: 2.62±1.36	<u>3 months</u><br>Non-diabetic and PD: 1.98±1.31<br>Well controlled diabetes and PD: 2.57±1.01<br>Poorly controlled diabetes +PD: 2.39±1.27	There was a decrease in CAL across both groups, with the Non-diabetic group reporting a slightly higher level of decrease at the 3 month interval.
Rode et. al, 2021	Mean CAL (mm) ± SD	Non-PD: 0	<u>3 months</u><br>Non-PD:0	There was a decrease in both groups, with the Diabetic group reporting a

		Non-diabetic and PD: 5.28 ± 0.80<br>T2DM and PD: 7.14 ± 1.59	Non-diabetic and PD: 3.12 ± 0.77<br>T2DM and PD:4.51 ± 1.38	slightly higher level of decrease at the 3 month interval.
Silva-Boghossian et. al, 2014	Mean CAL (mm) ± SD	Non- diabetic + PD: 4.3 ± 1.0<br>Diabetic and PD:4.9 ± 1.5	<u>3 months</u><br>Non-diabetic and PD: 3.9 ± 0.8<br>Diabetic and PD: 4.3 ± 1.3	There was a similar level of decrease in both groups at the 3 month interval.
Wang et. al, 2023	Mean CAL (mm) ± SD	Non-diabetic and PD: 2.9 ± 1.4<br>Diabetic + PD: 3.5 ± 0.6	<u>3 months</u><br>Non-diabetic and PD: 2.4 ± 1.0<br>Diabetic and PD: 3.1 ± 0.6	There was a similar level of decrease reported at the 3 month interval across both groups.
**Assessed prevalence of periodontal attachment loss (PAL) %**				
Christgau et. al, 1998	PAL 1-3mm as a median % per patient (25%/75%)	Non-diabetic and PD: 41.5<br>Diabetic and PD: 39.7	<u>2 weeks</u><br>Non-diabetic: 40.1<br>Diabetic: 43.9<br><u>4 months</u><br>Non-diabetic and PD:43.2<br>Diabetic and PD: 50.6	There was a similar level of decrease in CAL reported for both groups at all intervals.
	PAL 4-5mm as a median % per patient (25%/75%)	Non-diabetic and PD: 35.3<br>Diabetic and PD: 33.4	<u>2 weeks</u><br>Non-diabetic:35.3<br>Diabetic: 32.8<br><u>4 months</u><br>Non-diabetic and PD:38.5<br>Diabetic and PD:36	
	PAL ≥6mm as a median % per patient (25%/75%)	Non-diabetic and PD: 20.7<br>Diabetic and PD: 16.6	<u>2 weeks</u><br>Non-diabetic:19.7<br>Diabetic:13.7<br><u>4 months</u><br>Non-diabetic and PD:14.6<br>Diabetic and PD:9.4	

PD, periodontitis; ND, non-diabetic; DM, diabetes mellitus; T1DM, type 1 diabetes mellitus; T2DM, type 2 diabetes mellitus; CAL, clinical attachment loss/level; PAL, probing attachment level. Mean CAL (mm) ± SD indicates mean clinical attachment loss in millimetres with standard deviation. PAL categories (< 4 mm, 4–6 mm, ≥ 6 mm) and median percentages per patient (25th/75th percentiles) represent the proportion of sites within each attachment-level band.

Supplementary Table S9. Summary of changes in bleeding on probing following non-surgical periodontal therapy

Author, Year	BOP measure unit	Baseline	Post NSPT change	Overall Summary
Almeida et. al, 2019	BOP (%)	Non-diabetic and PD: 40.9 ± 18.9<br>Diabetic and PD: 45.5 ± 22.4	<u>3 months</u><br>Non-diabetic and PD: 27.2 ± 12.6<br>Diabetic + PD: 30.3 ± 15.3<br><u>6 months</u><br>Non-diabetic + PD: 28.1 ± 12.9<br>Diabetic + PD: 30.6 ± 18.7<br><u>12 months</u><br>Non-diabetic + PD: 22.4 ± 13.3<br>Diabetic + PD: 29.1 ± 15.6	Following NSPT, there was a decrease in BOP for both groups. The non-diabetic group reported a slightly greater decrease when comparing baseline to 12 months. The BOP for the non-diabetic group increased at 6 months compared to 3 months, however this value decreased again at the 12-month interval and was still decreased compared to baseline values.
Buzinin et. al, 2014	Bleeding Score	Non-diabetic and PD: 34.04 ± 20.56<br>Diabetic + PD: 36.7 ± 22.22	<u>2 months</u><br>Non-diabetic + PD: 17.52 ± 10.74<br>Diabetic + PD: 19.35 ± 10.81<br><u>3 months</u><br>Non-diabetic + PD: 11.76 ± 9.69<br>Diabetic + PD: 12.15 ± 7.68	Following NSPT, there was a decrease in BOP for both groups and both groups reported a similar level of decrease at all intervals.
Devina et. al, 2024	BOP (%)	Non-diabetic + PD (placebo gel): 56.0 ± 17.84<br>Non-diabetic + PD (hyaluronic acid gel): 44.89 ± 18.811<br>Diabetic + PD (placebo gel): 53.22 ± 30.298<br>Diabetic + PD (hyaluronic acid gel): 63.56 ± 25.125	<u>4 weeks</u><br>Non-diabetic + PD (placebo gel): 26.22 ± 13.645<br>Non-diabetic + PD (hyaluronic acid gel):<br>20.22 ± 17.782<br>Diabetic + PD (placebo gel): 27.67 ± 21.071<br>Diabetic + PD (hyaluronic acid gel): 35.22 ± 19.344	Following NSPT, there was a decrease in BOP for all groups with all groups reporting a similar level of decrease at the 4-week interval.
Duarte et. al, 2018	BOP (%)	Non-diabetic + PD:<br>61.3 ± 22.5<br>Diabetic + PD:	<u>3 months</u><br>Non-diabetic + PD: 15.1 ± 17.0<br>Diabetic + PD: 9.7 ± 4.9	Following NSPT, there was a decrease in BOP for both groups and the non-

		40.7 ± 14.0	6 months<br>Non-diabetic + PD: 13.5 ± 12.4<br>Diabetic + PD: 9.4 ± 5.2<br>12 months<br>Non-diabetic + PD: 12.5 ± 21.4<br>Diabetic + PD: 10.6 ± 5.7	diabetic group reported a slightly higher level of decrease at all intervals.
Hropot Plesko et. al, 2021	BOP (%)	Non-diabetic + PD: 57.52 ± 4.94<br>T1DM + PD: 53.30 ± 4.99<br>T2DM + PD: 39.80 ± 4.89	3 months<br>Non-diabetic + PD: 34.81 ± 4.76<br>T1DM + PD: 45.53 ± 4.98<br>T2DM + PD: 35.22 ± 4.77	Following NSPT, there was a decrease in BOP for both groups and the non-diabetic group reported a slightly higher level of decrease at the 3 months interval.
Silva-Boghossian et. al, 2014	BOP (%)	Non-diabetic + PD: 76.1 ± 16.1<br>Diabetic + PD: 90.9 ± 9.9	3 months<br>Non-diabetic + PD: 15.8 ± 10.7<br>Diabetic + PD: 28.2 ± 15.9	Following NSPT, there was a decrease in BOP for both groups and both groups reported a similar level of decrease at the 3-month interval.

Data are presented as mean ± standard deviation (SD). Time points refer to months or weeks post-NSPT as indicated in the table.
PD, periodontitis; ND, non-diabetic; DM, diabetes mellitus; T1DM, type 1 diabetes mellitus; T2DM, type 2 diabetes mellitus; BOP, bleeding on probing. BOP (%) denote the percentage of periodontal sites that bled on gentle probing. “Bleeding score” reflects a study-specific composite bleeding index reported as a percentage

Supplementary Table S10. Summary of changes in plaque levels following non-surgical periodontal therapy

Author Name and Year	Unit of measurement	Baseline	Post NSPT change
Almeida et. al, 2019	Sites with plaque accumulation (%)	Non-diabetic: 46.4 ± 19.1<br>Diabetic: 62.3 ± 26.8	<u>3 months</u><br>Non-diabetic: 31.8 ± 21.3<br>Diabetic: 41.3 ± 22.7<br><u>6 months</u><br>Non-diabetic: 31.2 ± 19.7<br>Diabetic: 36.5 ± 19.8<br><u>12 months</u><br>Non-diabetic: 23.4 ± 18.6<br>Diabetic: 36.4 ± 15.8
Buzinin et. al, 2014	Plaque score – visible plaque index	Non-diabetic: 32.4 ± 20.60<br>Diabetic: 45.85 ± 27.39	<u>2 months</u><br>Non-diabetic: 13.09 ± 11.3<br>Diabetic: 14.85 ± 9.37<br><u>3 months</u><br>Non-diabetic: 9.8 ± 5.86<br>Diabetic: 10.80 ± 10.81
Hropot Plesko et. al, 2021	FMPS (%)	Non-diabetic: 52.70 ± 13.06<br>T1DM: 54.34 ± 15.80<br>T2DM: 45.64 ± 20.80	<u>3 months</u><br>Non-diabetic: 33.27 ± 9.86<br>T1DM: 48.91 ± 12.86<br>T2DM: 34.16 ± 16.63
da Cruz et. al, 2008	PI	Non-diabetic: 91.33 ± 12.94<br>Diabetic: 90.24 ± 16.41	<u>1 month</u><br>Non-diabetic: 19.12 ± 10.14<br>Diabetic: 28.48 ± 14.86<br><u>3 months</u><br>Non-diabetic: 6.01 ± 2.66<br>Diabetic: 7.21 ± 2.98
Mirnić et. al, 2021	PI	Non-diabetic: 1.32 ± 0.51<br>Well controlled diabetes: 1.74±0.48<br>Poorly controlled diabetes: 1.88±0.37	<u>3 months</u><br>Non-diabetic: 0.66±0.49<br>Well-controlled diabetes: 1.14±0.46

			Poorly controlled diabetes:<br>1.31±0.37
Wang et. al, 2023	PI	Non-diabetic: 2.5 ± 0.5<br>Diabetic: 2.4 ± 0.7	<u>3 months</u><br>Non-diabetic: 2.5 ± 0.5<br>Diabetic: 1.4 ± 0.5
Duarte et. al, 2018	Plaque accumulation (% sites)	Non-diabetic: 77.5 ± 16.0<br>Diabetic: 77.8 ± 21.0	<u>3 months</u><br>Non-diabetic: 40.0 ± 24.5<br>Diabetic:30.2 ± 17.5

All data are presented as mean ± standard deviation (SD). PD, periodontitis; T1DM, type 1 diabetes mellitus; T2DM, type 2 diabetes mellitus; FMPS, full mouth plaque score; PI, plaque index; NSPT, non-surgical periodontal therapy. Sites with plaque accumulation (%)", FMPS (%), "plaque score – visible plaque index" and "plaque accumulation (% sites)" represent the proportion of tooth sites with visible plaque. PI values are study-specific plaque indices (Silness–Löe or modified versions) reported as mean scores per patient.

Supplementary Table S11. GRADE summary of findings: non-surgical periodontal therapy in adults with periodontitis with and without diabetes

Outcome	No. of studies / participants*	Study design	Overall findings (diabetic vs non-diabetic after NSPT)	Key limitations (downgrading domains)	Certainty of evidence (GRADE)
Microbiological outcomes (key pathogens / complexes)	11 studies; ~500–550 participants	Mix of RCTs and non-randomised	Directionally consistent reductions in major periodontal pathogens (e.g. *P. gingivalis, T. forsythia, A. actinomycetemcomitans)* in both groups after NSPT; qualitative evidence that diabetics generally respond similarly.	Serious heterogeneity in methods (culture, PCR, checkerboard, 16S), metrics (counts, % positive sites, proportions), and reporting; limited use of modern sequencing, very few alpha-diversity data and no beta-diversity analyses.	⊕⊕◯◯ Low
Periodontal probing depth (PPD)	11 studies; ~600–700 participants	5 RCTs, 6 non-randomised studies	All studies reported reduced mean PPD or % of deep pockets in both diabetic and non-diabetic groups after NSPT; most showed similar magnitude of reduction, with some favouring non-diabetics and some diabetics.	Risk of bias (several non-randomised studies; "some concerns" in RCTs), inconsistency in effect size and follow-up intervals, indirectness (different indices, thresholds), imprecision (no pooled estimates).	⊕⊕⊕◯ Moderate
Clinical attachment loss/level (CAL / PAL)	11 studies; ~600–700 participants	5 RCTs, 6 non-randomised studies	All studies found reductions in mean CAL or PAL categories in both groups; most reported comparable gains, with a few showing slightly greater CAL improvement in non-diabetics or, less often, in diabetics.	Similar concerns to PPD: mixed designs with some moderate/serious ROBINS-I ratings, heterogeneous reporting (mm vs % sites; different cut-offs), limited statistical comparisons ND vs DM, no meta-analysis.	⊕⊕⊕◯ Moderate
Bleeding on probing (BOP)	6 studies; ~300–350 participants	3 RCTs, 3 non-randomised studies	All studies reported decreased BOP percentages after NSPT in both diabetic and non-diabetic participants; most showed similar	Risk of bias (non-randomised designs; "some concerns" in several trials), inconsistency in indices and timing, occasional	⊕⊕⊕◯ Moderate

			reductions, with a few studies indicating slightly greater reduction in non-diabetics.	transient increases (e.g. 6-month BOP in one study), limited formal between-group testing.	
Plaque scores (plaque index / % sites with plaque)	6–7 studies; ~350–400 participants	Mix of RCTs and non-randomised	All studies found reductions in plaque scores or % plaque-positive sites in both groups following NSPT; no consistent difference between diabetic and non-diabetic groups was evident.	Overall high/unclear risk of bias for plaque as a secondary outcome, heterogeneous plaque indices, sparse reporting of variability and p-values, no pooled effect sizes or prespecified minimal important difference.	⨁⨁◯◯ Low
Alpha diversity of the subgingival microbiome	1 study; 22 participants	Non-randomised parallel study	One 16S rRNA study reported an increase in alpha diversity following NSPT in both diabetic and non-diabetic participants, with no clear difference between groups.	Single small study at serious risk of bias; indirect (specific population, short follow-up), no replication or pooled estimate, no data on clinical impact threshold.	⨁◯◯◯ Very low

**Participant numbers are approximate, based on total sample sizes across included studies; exact denominators vary slightly by outcome.*
RCT, randomised controlled trial; ND, non-diabetic; DM, diabetes mellitus; NSPT, non-surgical periodontal therapy; PPD, periodontal probing depth; CAL, clinical attachment loss; PAL, probing attachment level; BOP, bleeding on probing; PCR, polymerase chain reaction; 16S rRNA, 16S ribosomal RNA sequencing; ROBINS-I, Risk Of Bias In Non-randomised Studies - of Interventions.

GRADE certainty of evidence: ⨁⨁⨁⨁ High = further research is very unlikely to change our confidence in the estimate of effect; ⨁⨁⨁◯ Moderate = further research is likely to have an important impact on our confidence in the estimate of effect and may change the estimate; ⨁⨁◯◯ Low = further research is very likely to have an important impact on our confidence in the estimate of effect and is likely to change the estimate; ⨁◯◯◯ Very low = we are very uncertain about the estimate.

Supplementary Figures

Supplementary Figure S1. Cochrane Risk of Bias (RoB) Tool for Randomised Trials (RoB 2) Summary

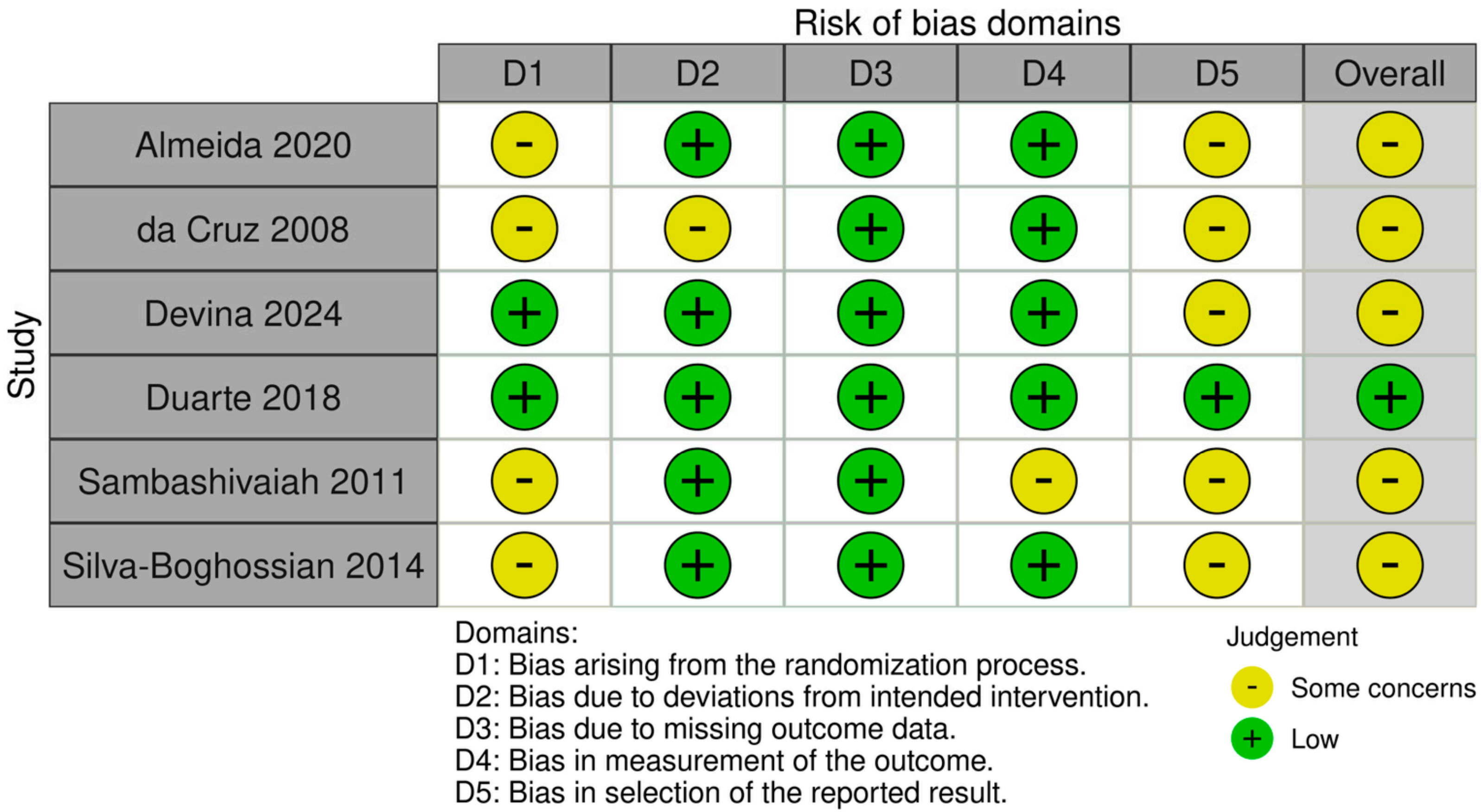

Supplementary Figure S2. Cochrane Risk of Bias in Non-randomised Studies – of Interventions (ROBINS) Tool Summary

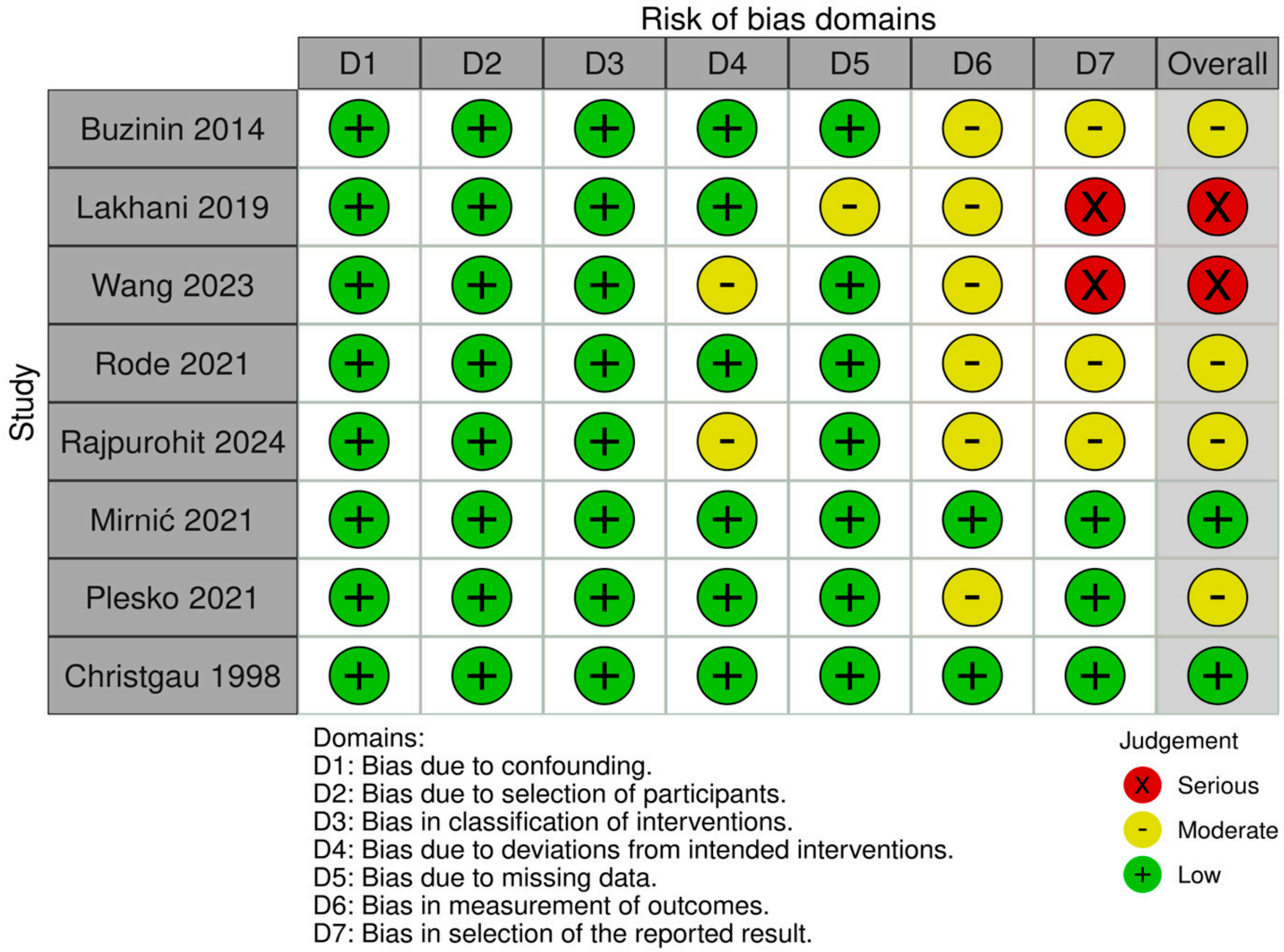

Supplement: Supplementary file 1 [file dentistry-14-00617-s001.zip › dentistry-4521201-supplementary.pdf]
